# Fish telocytes and their relation to rodlet cells in ruby-red-fin shark (rainbow shark) *Epalzeorhynchos frenatum* (Teleostei: Cyprinidae)

**DOI:** 10.1038/s41598-020-75677-3

**Published:** 2020-11-03

**Authors:** Hanan H. Abd-Elhafeez, Walied Abdo, Basma Mohamed Kamal, Soha A. Soliman

**Affiliations:** 1 Department of Anatomy, Embryology and Histology, Faculty of Veterinary Medicine, Assiut University, Assiut, 71526 Egypt; 2 Department of Pathology, Faculty of Veterinary Medicine, Kafrelsheikh University, Kafr el-Sheikh, 33516 Egypt; 3 Anatomy and Embryology Department, Faculty of Veterinary Medicine, University of Sadat City, Sadat City, 32897 Egypt; 4 Department of Histology, Faculty of Veterinary Medicine, South Valley University, Qena, 83523 Egypt

**Keywords:** Cell biology, Immunology, Stem cells

## Abstract

Telocytes comprise the major constituents of the supportive interstitial framework within the various organs. They form a 3D network between different types of stromal and non-stromal cells, which makes them distinctively vital. We have previously explored the origin of the peculiar rodlet cells, especially on their differential stages in aquatic species. The current study aimed at highlighting the relation of telocytes with different rodlet stages. Samples of fish, olfactory organs, and gills were processed for semi thin sections, transmission electron microscopy, and immunohistochemistry. It was evident in the study that telocytes formed a 3D interstitial network, entrapping stem cells and differentiating rodlet cells, to establish direct contact with stem cells. Differentiated stem cells and rodlet progenitor cells, practically in the granular and transitional stages, also formed ultrastructure junctional modifications, by which nanostructures are formed to establish cell contact with telocytes. Telocytes in turn also connected with macrophage progenitor cells. Telocytes (TCs) expressed CD34, CD117, VEGF, and MMP-9. In conclusion, telocytes established direct contact with the stem and rodlet cells in various differential stages. Telocytes may vitally influence stem/progenitor cell differentiation, regulate rodlet cell function, and express MPP-9 that may regulate immune cells functions especially, including movement and migration ability.

## Introduction

Telocytes are considered a vital component of the stromal framework of various organs. Their morphological criteria allow communication with different types of stromal and non-stromal cells. They are comprised of cell bodies from which several cell prolongations emerge, which are called telopodes, later forming a 3D labyrinthine network structure, which widely spreads between cellular and non-cellular elements. Telopodes have alternating thin segments called podomers and interval expansions called podoms that are richly found in mitochondria, the endoplasmic reticulum, and caveolae^1^.

Telocytes have a major function of affecting other cells, either by establishing cellular contact or paracrine signaling. This cellular contact involves both homocellular and heterocellular forms of contact. It was found that various forms of these heterocellular contacts are noticeable between telocytes and other cells, such as minute junctions, point contacts, nano contacts planar contacts, and paracrine singaling^2^. Different forms of cell contact are described such as direct apposition of the cell membrane of adjacent telocytes, adherence (puncta adherents minima, process adherents, and manubrium adherents), and gap junction. The latter form, the gap junction is significant in signal transduction between cells^2,3^. Telocytes transmits microvesicles and macromolecules, such as proteins or RNAs, and microRNAs, to the recipient cells. Moreover, they secrete different types of extracellular vesicles, including exosomes, ectosomes and multivesicular vesicles^1,4,5^.

One of the most powerful roles of telocytes, aside from supplying a structural framework, is to organize the functional support to tissues and organs. Telocytes naturally trigger muscular contraction by the exertion of nerve impulses^6–9^. Telocytes have been found to exhibit receptors for both excitatory and inhibitory neurotransmitters^10^. It was also discovered that telocytes are considered mechanoreceptors and might actually promote atrial fibrillation^11^. The wide functional significance of telocytes has been categorized in terms of which genes they express. A number of studies found that telocytes and their function largely contribute to cellular signaling^12,13^; tissue homeostasis, remodeling^13^; and repair^14^; cell expansion and movement^12^; angiogenesis^13^; embryogenesis^15^; morphogenesis^16^; protection from oxidative agents and prevention of cellular aging^17^; and suppression of inflammation and oncogenesis^18^.

Rodlet cells are special cells noted in both fresh and marine fish^19^. They are widely distributed throughout the fish body. They are particularly found in the respiratory organs; the digestive, genital, integument, immune, cardiovascular, and skeletal system; the eye; and the abdominal cavity^20–22^. Many functions have been deemed for rodlet cells such as ion transportation, osmoregulation^23^, and sensory function^24^. Interestingly, studies have also agreed that the rodlet cell has a key role in the immune defense of fish, and, therefore it may be considered as a type of granulocytes^25–27^. Also of note is that these cells have a secretory function^21,28^ with a holocrine mode of secretion^21^.

Several investigations support the hypothesis that telocytes serve a valuable role especially in tissue and organ regeneration. The telocytes’ potential in tissue renewal has been associated with its role in the skeletal muscle, skin, heart, lung, liver, uterus, urinary system, meninges and choroid plexus, and eye^14^. The previous study recognized the origin of rodlet cells and described their sequential differentiation in ruby shark. Rodlet cell progenitors are located in the olfactory stroma. They are characterized by extensive vesicular contents, at the vesicular stage. They later transform to granular rodlet cells, which are found to consist of immature rodlet granules. The transitional rodlet cells develop small rodlet granules that have a central rod core. The mature rodlet cells then consist themselves of typical elongated rodlet granules^21^.

The current study is aimed at investigating the telocytes’ relation to stem cells, macrophage, and rodlet precursors in ruby Shark. Ruby shark is a member of the family *Cyprinidae* a fish species mainly characterized by localization of rodlet cells^29,30^. We used different conventional histological and immunohistochemistry staining and semi thin sections for light microscopy, scanning electron microscopy (SEM), and ultrathin sections for transmission electron microscopy (TEM) for the identification of telocytes, stem cells, rodlet, and macrophage progenitor cells.

## Materials and methods

### Ethical approval

The ethics committee of Assiut University, and veterinary authorities in Assiut province, Egypt approved the method of the work. "All methods were performed in accordance with the relevant guidelines and regulations".

### Sample collection

Fish were obtained from an ornamental shop in Assiut City, Egypt. All fish were anesthetized using benzocaine (4 mg/L). The fish measured 10 to 12 cm in standard body length. Samples were taken from apparently healthy fish; ruby-red-fin Shark, (rainbow Shark), *Epalzeorhynchos frenatum *(Teleostei: Cyprinidae). Four samples were used for light microscopic examination. Separately, three of the samples were used for normal paraffin embedding and immunohistochemistry study, and one sample was osmicated using osmium tetroxide before paraffin embedding. Two more samples were used for the scanning electron microscopic study, and another two were processed for semi thin sectioning.

### Sample fixation and processing

#### Light microscopic examination

The whole fish is immersed in a mixture of 20 mL of 2.5% glutaraldehyde and 80 mL 0.1 M Na-phosphate buffer (pH 7.2–7.4). Samples were processed according to Abdelhafeez and Soliman^31^ as the following: washed samples were immediately fixed after excision in Bouin’s fluid for 2 h. To get rid of the fixative before the subsequent processing, the fixed samples were thoroughly washed using 70% ethanol (3 × 24 h). The samples were then cleared in methyl benzoate and embedded in paraffin wax for 8 h. Serial transverse sections and longitudinal sections were cut at 5 µm by using a Richert-Leica RM2125 Microtome, Germany. For general histological examination, the paraffin representative sections were stained by hematoxylin and eosin satin^32^.

#### Histochemical investigation

The following histochemical stains were used to identify telocyte staining affinity in the study: trichrome according to Crossomon’s^33^ and Mallory’s triple trichrome stain^34^, combined Alcian blue pH 2.5 periodic acid Schiff (AB pH 2.5/PAS) techniques^35^, safranin O^36^. Other histochemical stains used were the Wiegert^37^ and Van Gieson^38^ counterstain, Grimelius’s silver nitrate method^36^, and Heidenhain’s iron-Hx^39^. Protocols of all staining were cited by^36^ The obtained stained slides were examined with a Leitz Dialux 20 microscope provided with a Canon (Power shot A95) digital camera.

#### Osmium tetroxide paraffin procedure for fat

The collected gills were placed in osmium tetroxide after fixation in 10% neutral buffer formalin (NBF) for demonstration of fat by a method that allows for tissue to be paraffin-embedded^40^.

Sample osmication was performed using the following reagents: 1% osmium tetroxide solution; osmium tetroxide and distilled water and 0.5% periodic acid solution; and periodic acid and distilled water. The procedure start by trimming the formalin fixed tissue to 2 mm thick, then washing the tissue thoroughly for at least 30 min. Then the tissue is rinsed well in distilled water and kept in a small quantity of osmium tetroxide solution for at least 1 to 2 h. The slides are washed again twice in distilled water for 15 min. The slides are differentiated by placing them in the 0.5% periodic acid solution for 30 min, followed by washing in tap water for 30 min. The osmicated samples then are processed for the preparation of paraffin blocks. Routinely processing of the tissue samples began with dehydration in alcohols, clearing, and then embedding as usual. Four-to five-microns-thick sections prepared from paraffin blocks were then picked on slides then dried and were deparaffinized and hydrated as usual. Sections were counterstained using hematoxylin staining, dehydrated, cleared, and mounted on slides.

### Sudan black B on paraffin section^36^

Sudan black B (2–3 g) was dissolved in 100 ml 70% warm alcohol. The stain is placed at 60 °C for 3–4 h, then left to be cooled and filtrated. Sudan black stain was applied to the deparaffinized sections overnight; then, the sections were washed by DW, mounted with glycerin jelly. The solution was prepared by 15 g gelatin dissolved in 100 ml 0.2 M phosphate buffer (pH7) under moderate heat; then, 100 ml glycerol is added to the mixture.

### Acridine orange (fluorescent stain)^41^

The procedure was performed according to the procedures used by of Hoff et al. modified in^42–47^.

Acridine Orange is a cationic dye and stains protein-containing membranous vesicles including secretory vesicles, membrane-bounded acidic compartments, and lysosomes are acidic in nature. The compound exhibits a metachromatic reaction, which is associated with the liberation of green and red fluorescence. Acridine orange reacts with the membrane bounded vesicles, which makes it appear orange or red. Acridine orange is used for the identification of secretory vesicles and lysosomes^48–50^.

### Immunohistochemistry (IHC) staining of matrix metalloproteinase-9 (MPP-9)

We used a mouse anti-rabbit antibody against matrix metalloproteinase-9 (MPP-9). Immunohistochemical staining was performed on the paraffin sections of the whole fish section after choosing the sections containing gills, olfactory rosette using super frost plus microscope slides. Antigen localization was achieved using mouse anti-rabbit antibody against matrix metalloproteinase-9 (MPP-9) combined with the avidin–biotin complex (ABC) technique^51^ using the reagent of Ultra Vision Detection System (Anti-Polyvalent, HRP/DAB ready to use, Thermo Fisher Scientific TP-015HD), according to the manufacturer’s instructions. Briefly ,the procedures were done as follows^21,52^: paraffin sections of 5 µm in thickness were dewaxed by xylene, rehydrated by ascending grades of alcohol, and rinsed by PBS pH 7.4 (3 times for 5 min). For the suppression of endogenous peroxidase activity, the sections were placed in hydrogen peroxide blocks at room temperature. Thereafter, the sections were washed by running tap water for an additional 10 min. To enhance antigen retrieval, the slides were treated with a 10 mm sodium citrate buffer (pH 6.0) (Table 1) at 95–98 °C in a water bath for 20 min; then, the slides were cooled for 20 min at room temperature and were subsequently washed in PBS (pH 7.4, 3 times for 5 min). Blocking nonspecific background staining was performed by using ultra V block for 5 min at room temperature. Ultra V block application was preventing from exceeding 10 min to avoid staining artifact. The primary antibody (Table 2) was applied to the sections overnight at 4 °C. Mouse anti-rabbit antibody, the primary antibody used against matrix metalloproteinase-9 (MPP-9; RB-9423-PO, Thermo Fisher Scientific, UK; Lab Vision Corporation; at 1:25 dilution in PBS) applied to the section overnight at 4 °C; then, sections were washed using PBS (pH 7.4, 3 times for 5 min). The biotinylated secondary antibody (Goat Anti-Polyvalent, Anti-Mouse IgG + Anti-Rabbit IgG; Thermo Fisher Scientific, UK; Lab Vision Corporation; Table 2) was applied to sections for 10 min at room temperature. Then, sections were washed by PBS (pH 7.4, 3 times for 5 min) and subsequently incubated with streptavidin- peroxidase complex (Thermo Fisher Scientific, UK; Lab Vision Corporation, USA) for 10 min at room temperature. Visualization of the bound antibodies was performed using 1 drop of DAB plus chromogen to 2 mL of DAB plus substrate. The mixture was applied and incubated at room temperature for 5 min. The incubation processes were carried out in a humid chamber. Harris hematoxylin was used as counterstains for 30 s. The sections were dehydrated using ethanol and isopropanol I and II, cleared in xylene and covered by DPX. Immunohistochemical staining was examined using the Leitz Dialux 20 microscope provided with the canon (Power shot A95) digital camera.

**Table 1:** Components of the fixative.

Fixative	Components	Amount
Karnovsky fixative	Paraformaldehyde, 25% freshly prepared	10 ml
	Glutaraldehyde 50%	10 ml
	Na-phosphate buffer (0.1 M, pH 7.4)	50 ml
	Distilled water	30 ml
Na-phosphate buffer (0.1 M, pH 7.4)	Solution A	
	Na_2_HPO_4_ 2H_2_O	17.02 g
	Distilled water	600 ml
	Solution B	
	NaH_2_PO_4_ H_2_	6 g
	Distilled water	200 ml
	Using solution	
	Solution A	580 ml
	Solution B	219 ml
Citrate-buffer (pH 6.0)	Solution A	
	Citrate C_6_H_8_O_7_ H_2_O	21 g
	Distilled water	1 L
	Solution B	
	Sodium citrate Na_3_C_6_H_5_O_7_ 2H_2_O	29.41 g
	Distilled water	1 L
	Using solution	
	Solution A	9 ml
	Solution B	41 ml
	Distilled water	Add 500 ml

**Table 2:** Identity, sources, and working dilution of antibodies used in immunohistochemical studies.

Target	Primary antibody supplier	Origin (catalog no)	Dilution	incubation	Antigen retrieval	secondary antibody-incubation time
CD34	Rat anti-mouse CD34 antibody ( e bioscience, San Diego, CA	Mouse CD34 monoclonal antibody (clone: RAM34) (Cat.no 14–0341-85)	1:100	Over night	Boiling in citrate buffer (pH 6.0), 20 min Goat	Goat anti-mouse IgG (H + L) secondary antibody Catalog # 31569 Dilution ; 1:100 One hour at room temperature
CD117	Anti-CD117 (e Bioscience, San Diego, CA)	Mouse Cd117 monoclonal antibody (clone. ACK2) (Cat. no. 14–1172–82)	1:100	Over night	Boiling in citrate buffer (pH 6.0), 20 min	
VEGF	Anti-VEGF rabbit monoclonal antibody boster biological technology	Rabbit Monoclonal (clone:ADB-22) CA 94,566	1:100	Over night	Boiling in citrate buffer (pH 6.0), 20 min	Goat anti-rabbit secondary antibody (cat. no. K4003, EN Vision + TM System Horseradish Peroxidase Labelled Polymer; Dako) Ready to use 30 min at room temperature
MPP-9	Anti-MPP9 Thermo Fischer Scientific, Lab vision Corporation, Fremont, USA	Mouse (mc, Ab-1) Clone D(33)376 RB-9423-PO Rabbit polyclonal	1:30	Over night	Boiling in citrate buffer (pH 6.0), 20 min	Biotinylated goat Anti-Polyvalent, Anti-mouse Igg + Anti-Rabbit Igg, Thermo Fisher Scientific, The UK. Lab Vision Corporation; USA Ready to use One hour at room temperature

Antibodies used that showed reactivity in fish species.

### Immunohistochemical procedures of VEGF, CD34, and CD117

Two-step immunohistochemical staining technique was used using the DAKO EN Vision TM + System, HRP peroxidase^53^.

The procedure of staining was done according Abdo et al.^54^. Briefly, sections 5 µm thick paraffin-embedded sections were dewaxed, rehydrated, and rinsed in PBS pH 7.4 (3 times for 5 min). Endogenous peroxidase was inhibited by adding drops of 3% hydrogen peroxide in methanol at room temperature for 20 min, followed by intense washing under running tap water for an additional 10 min. For antigen retrieval, slides were placed in a 10-mm sodium citrate buffer (pH 6.0) (Table 1) and heated to 95–98 °C in a water bath for 20 min followed by cooling for 20 min at room temperature. Sections were then rinsed in PBS (pH 7.4, 3 times for 5 min). Sections were covered by adding drops of blocking serum (DAKO) to cover the sections for 5 min at room temperature to block nonspecific background staining (note: application of blocking should not exceed 10 min or there may be a reduction in the desired stain). Sections were then incubated with the primary antibody (Table 2, identity, sources, and the working dilution of antibodies used in immunohistochemical studies). After incubation, slides were washed with PBS (pH 7.4, 3 times for 5 min), followed by incubation for 30 min at room temperature with secondary antibody at room temperature. The slides were thereafter rinsed in PBS (pH 7.4, 3 times for 5 min) followed by incubation for 5–10 min at room temperature with 3,3′-diaminobenzidine (DAB) + substrate-chromogen which results in a brown-colored precipitate at the antigen site. The sections were counterstained with Harris hematoxylin for 30 s. The sections were dehydrated using ethanol alcohol 90%, and 100% II, cleared in xylene, and covered by DPX. Immunohistochemical staining examined by using the Leitz Dialux20 microscope provided with the Canon (PowerShot A95) digital camera.

Negative controls were carried out with the same procedure without using primary antibodies.

#### Electron microscopic examination

Two parts from olfactory organ and gills were carefully excised and fixed in Karnovsky’s fixative^55^ (10 ml paraformaldehyde 25%, 10 ml glutaraldhyde 50 %, 50 ml phosphate buffer, and 30 ml DW, Table 1) for scanning electron microscopy and transmission electron microscopy.

### Preparations of resin embedding samples for semi thin and ultra-thin sections

Small specimens from gills were used for semi thin sections. Small pieces 2.0–3.0 mm long were fixed in Karnovsky fixative^55^ at 4 °C overnight. They were processed according to the description of^21,56^. Samples were washed 4 times for 15 min in 0.1 Msodium phosphate buffer (pH 7.2). Samples were then post-fixated using 1% osmic acid in 0.1 M Na-phosphate buffer at 4 °C for 2 h then washed 3 times for 20 min in 0.1 Mphosphate buffer (pH 7.2). Dehydration through graded ethanol to propylene oxide was as follows: samples dehydrated in ethanol using the ascending grade of 50% for 30 min, 70% overnight, 90% for 30 min, 100% I for 30 min and 100% II for 60 min. Resin embedding was performed using propylene oxide (Merck, Darmstadt, Germany) for 30 min, epon: propylene oxide (1:1) (about 30 min), then in epon (for 3 h). Epon was prepared through mixing 5 ml Epon812 (Polysciences, Eppelheim, Germany) to 5 ml Araldite and 12 ml DDSA. Samples were embedded in epon and incubated at 60 °C. Samples thereafter were polymerized using epon mix and accelerator (DMP30) (1.5%). The blocks were incubated for 3 days as the follows: 60 °C on the first day, 70 °C on the second day , and 75 °C on the third day. Semi thin sections (1 µm) were cut using an ultra-microtome Ultracut E (Reichert-Leica, Germany) and stained with toluidine blue^57^.

Ultra-thin sections were obtained from semi thin section of gill by a Reichert ultra-microtome. The sections (70 nm) were stained with uranyl acetate and lead citrate^58^ and examined by JEOL100CX II transmission electron microscope at the Electron Microscopy Unit of Assiut University.

### Sample preparation for scanning electron microscopic (SEM) investigation

SEM samples were prepared according to^21^. The olfactory rosettes and the gills were excised from the head, washed using 0.1 M Na-phosphate buffer, and then fixed in Karnovsky’s fixative for 4 h at 4 °C. The specimens were washed before and after post-fixation in 1% osmic acid solution diluted in 0.1 M Na-phosphate buffer for 2 h at room temperature. Samples were dehydrated by alcohol 50%, 70%, and 90% for 30 min in each concentration and 100% for 2 days and then treated with isoamyl acetate for 2 days. Sample dryness was completed using a critical point drying method with a polaron apparatus. A JEOL − 1100 e ion sputtering device was used in gold-coating the samples. The samples were then examined by a JEOL scanning electron microscope (JSM_5400 LV) at KV10.

### Digital coloring scanning electron microscopic images

We digitally colored the transmission electron microscopic images using the Photo Filter 6.3.2 program to recognized different types of cells and structures. The methods were used by^21,31,59–70^.

### CMEIAS color segmentation: (for the supplementary images)

Negative images performed by using CMEIAS Color Segmentation is a free, improved computing technology used to process color images by segmenting the foreground object of interest from the background^71^. This has been done by the following steps: open image with CMEIAS Color Segmentation, then select “Process” from the menu items and subsequently choose “Negative image”^63,65,72^.

## Results

The current study investigated the localization of telocytes in the red-fin shark and their relation to stem cells and rodlet cells at different stages of development.

### Light microscopic findings

#### General and histochemical staining

**Figure 1:**
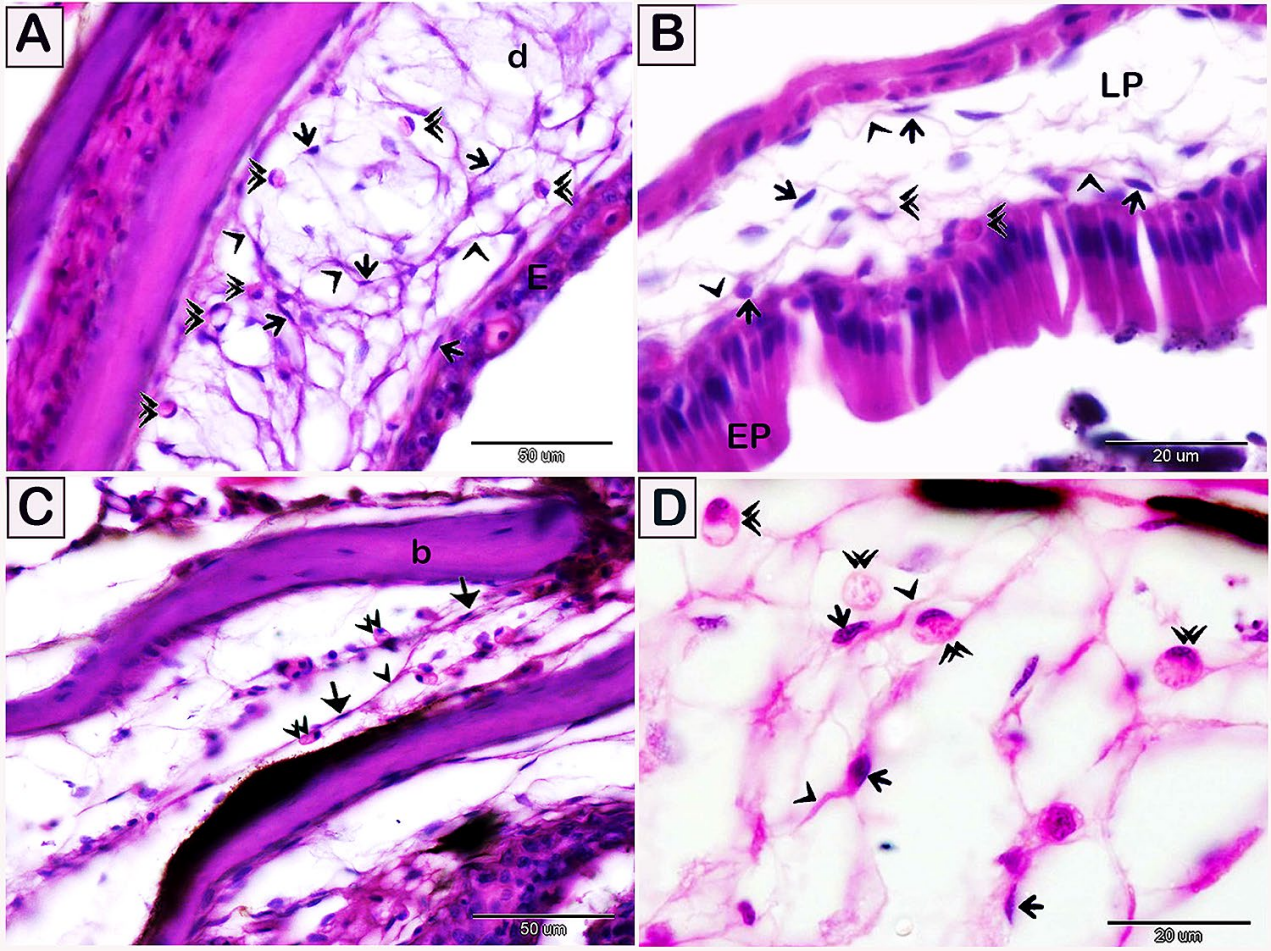
Visualization of telocytes in the skin, intestine, head, and gill arch of the red-fin shark using H&E. Paraffin sections of the skin (**A**), intestine (**B**), head between cephalic bone (**C**), and gill arch (**D**) stained by H&E. (**A**) Telocytes (arrows) located in the dermis (d). Telopodes (arrowheads) formed a 3D network in the dermis. (E, epidermis). Rodlet cells (double arrowheads). (**B**) Telocytes (arrows) located in the lamina propria (LP). Telopodes (arrowheads) formed a 3D network. (EP, intestinal epithelium) Rodlet cells (double arrowheads). (**C**) Telocytes (arrows) formed a 3D network between cephalic bones (b). Note telopodes (arrowhead). Rodlet cells (double arrowheads). (**D**) Telocytes (arrows) located in the submucosa of the gill arch. Telopodes (arrowhead) formed a 3D network. Note rodlet cells (double arrowheads). Magnification: (**A**,**C**) ×400; (**B**,**D**) ×1000.

**Figure 2:**
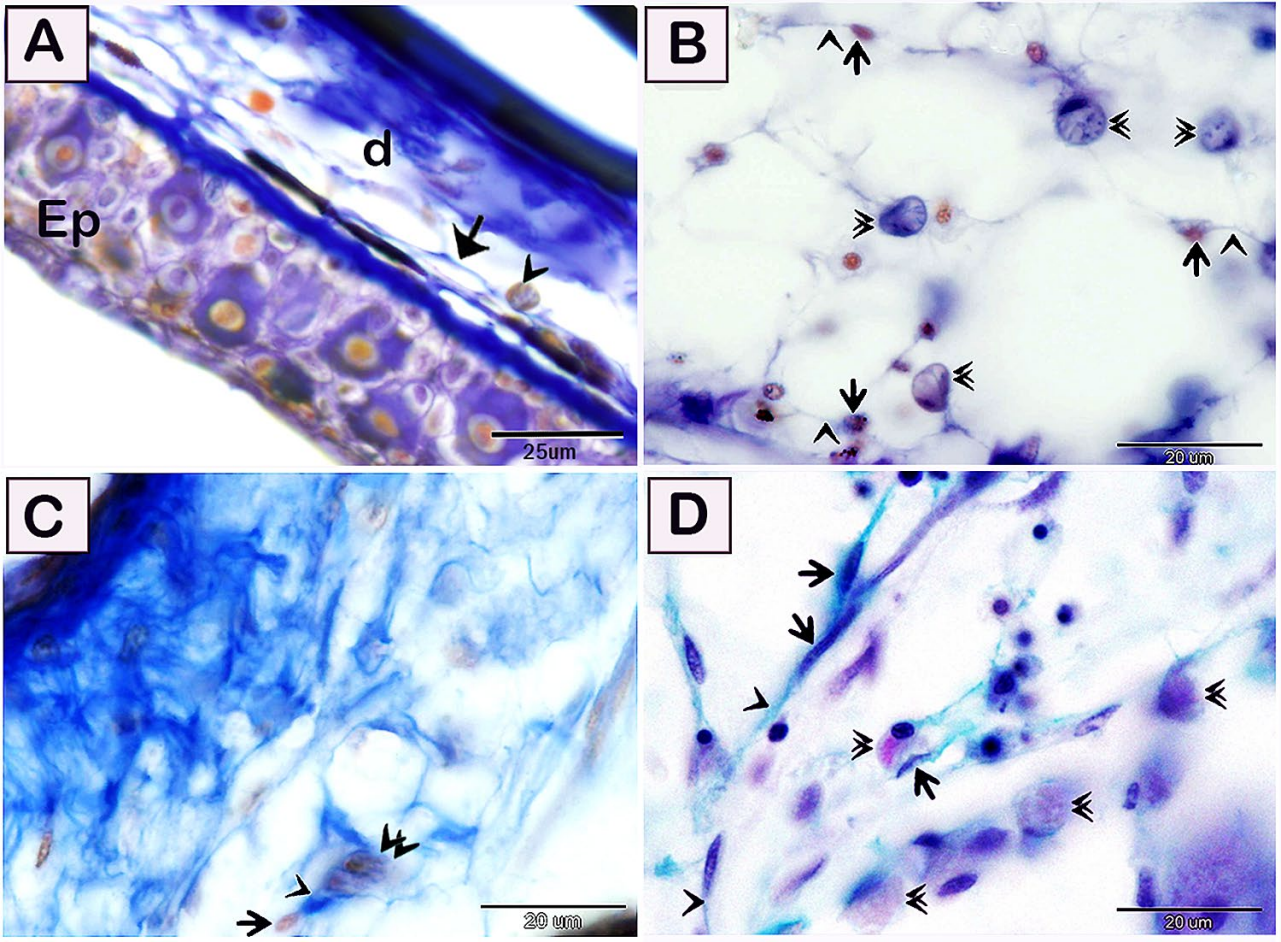
Visualization of telocytes in of the red-fin shark using trichrome stains. Paraffin sections of the skin (**A**) and gill arch (**B**–**D**) stained by the Mallory triple trichrome (**A**–**C**) and Crossomon’s trichrome (**D**). (**A**) Telocytes (arrows) located in the dermis (d). Note epidermis (EP) and rodlet cells (arrowhead). (**B**) Telocytes stained blue by the Mallory triple trichrome. Telocytes (arrows) located in the submucosa of the gill arch. Telopodes (arrowhead) forming a 3D network. Note Rodlet cells (double arrowheads). (**C**) Telocytes (arrows) in closed vicinity to the rodlet cells (double arrowheads). Note telopodes (arrowhead). (**D**) Telocytes (arrows) stained green by Crossomon’s trichrome formed a 3D network in the gill arch. Note telopodes (arrowhead), and rodlet cells (double arrowheads). Magnification: (**A**) ×400; (**B**–**D**) ×1000.

**Figure 3:**
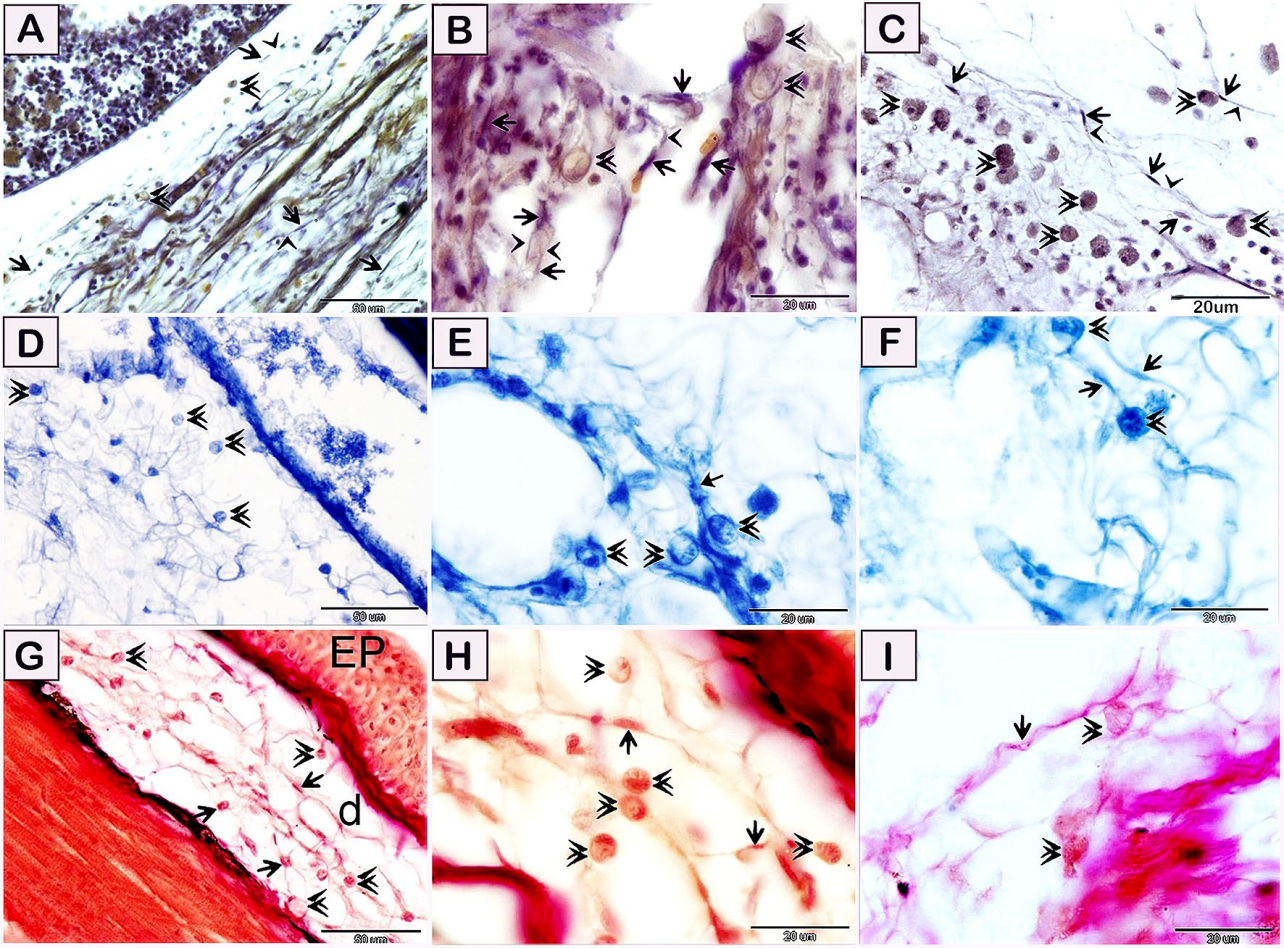
Visualization of telocytes in of the red-fin shark using osmic acid, iron hematoxylin, the Weigert Van Gieson method, and safranin O. Paraffin sections of the gill arch (**A**–**F**,**H**,**I**), skin (**G**) stained by osmic acid (**A**–**C**), iron hematoxylin (**D**–**F**), the Weigert Van Gieson method (**G**,**H**), and safranin O (**I**). (**A**–**C**) Telocytes stained brown by osmic acid. Telocytes (arrows) located in the submucosa of the gill arch. Telopodes (arrowheads) formed a 3D network. Note rodlet cells (double arrowheads). (**D**–**F**) telocytes (arrows) located in the submucosa of the gill arch. Telopodes formed a 3D network. Note rodlet cells (double arrowheads). Telocytes stained bluish black by iron hematoxylin. (**G**,**H**) Telocytes stained red by the Weigert Van Gieson method. Telocytes (arrows) located in the dermis (d). Telopodes (arrowheads) formed a 3D network in the dermis. Note epidermis (EP). Rodlet cells (double arrowheads). (**I**) Telocytes stained reddish orange by safranin O stain. Telocytes (arrows) located in the submucosa of the gill arch. Note rodlet cells (double arrowheads). Magnification: (**A**,**D**) ×400; (**B**,**C**) and (**E**–**I**) ×1000.

**Figure 4:**
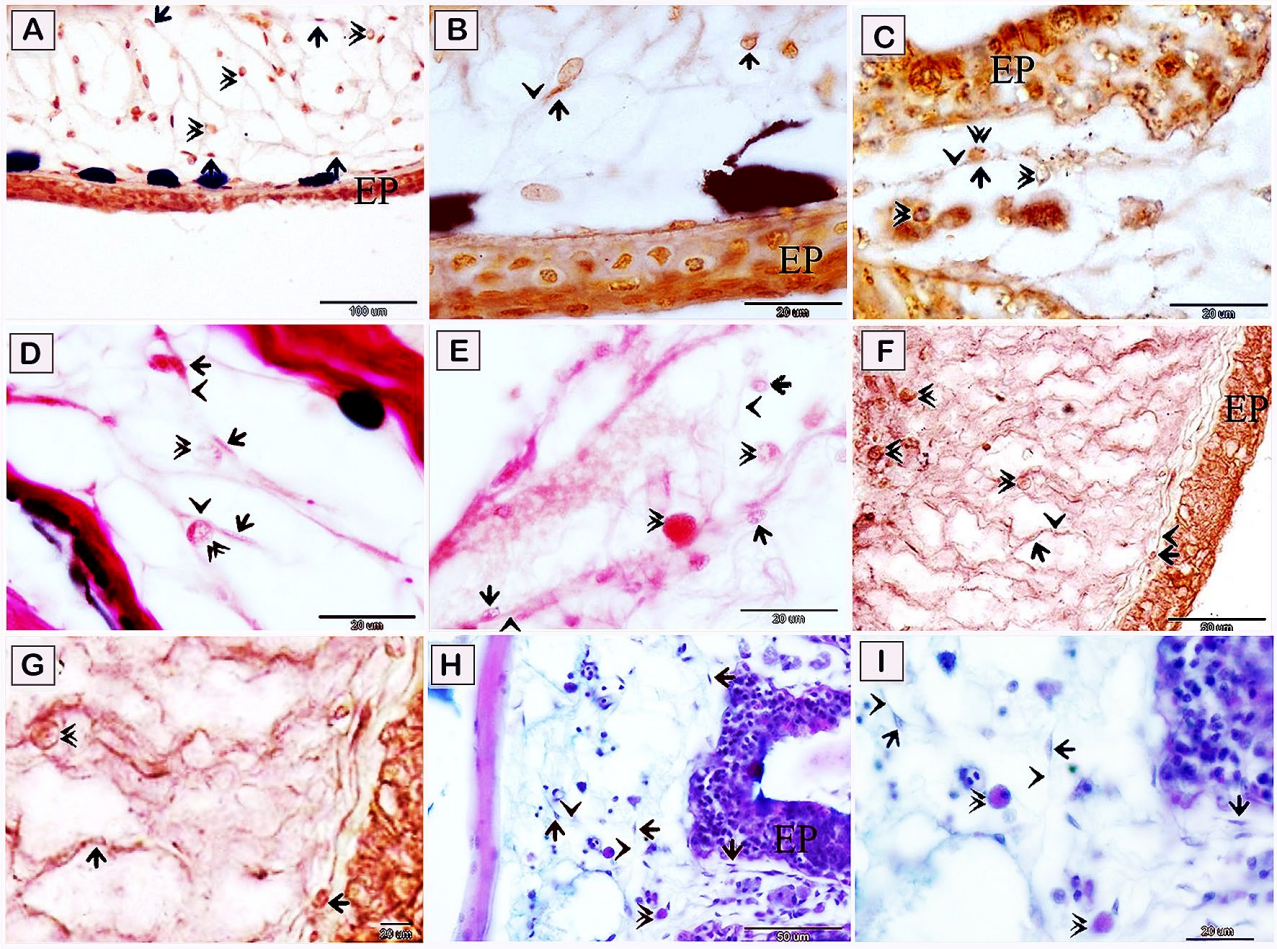
Visualization of telocytes of the red-fin shark using Grimelius’s silver nitrate method, the Wiegert’s Van Gieson method, Sudan black B, AB PH 2.5/PAS. Paraffin sections of the skin (**A**–**G**), submucosa of the olfactory rosette (**H**,**I**) stained by Grimelius’s silver nitrate method, (**A**–**C**), the Wiegert’s Van Gieson method (**D**,**E**), Sudan black. B (**F**,G), and AB pH 2.5 /PAS. (**H**,**I**). (**A**–**C**) Telocytes stained brown by Grimelius’s silver nitrate, telocytes (arrows) located in the dermis .Telopodes (arrowheads) formed a 3D network in the dermis. Note epidermis (EP), rodlet cells (double arrowheads). and epithelium (EP). (**D**,**E**) telocytes stained reddish orange by Van Gieson and (arrows) located in the dermis .Telopodes (arrowhead) formed a 3D network in the dermis. Note epidermis (EP). Rodlet cells (double arrowheads). (**F**,**G**) Telocytes stained brown by Sudan black B. (**H**,**I**) Telocytes (arrows) located in the submucosa of the olfactory rosette. and stained blue by Alcian blue pH 2.5/PAS. Telopodes (arrowheads) formed a 3D network. Note rodlet cells (double arrowheads). Epithelium (EP). Magnification: (**A**) ×200; (**H**) ×400; (**B**–**G**) and (**I**) ×1000.

Telocytes were identified by their telopodes using the light microscopic examination. They were located within the dermal tissue (Fig. 1A), gut mucosa (Fig. 1B), cephalic region (Fig. 1C), and submucosa of the gill arch (Fig. 1D). Telocytes established a 3D network that encloses numerous rodlet cells (Fig. 1A,C,D). Telocytes affinity for different histochemical staining methods has been observed: telocytes stained blue by the Mallory triple trichrome (Fig. 2A–C), green by Crossomon’s trichrome (Fig. 2D), brown by osmic acid (Fig. 3A–C),bluish black by iron hematoxylin (Fig. 3D–F), orange by the Van Gieson staining (Fig. 3G,H), reddish orange by safranin O (Fig. 3I), brown by Grimelius’s silver nitrate (Fig. 4A–C), red by the Wiegert’s Van Gieson method (Fig. 4D,E), brown by Sudan black (Fig. 4F,G), and blue by AB pH 2.5/PAS (Fig. 4H,I).

#### Semi thin technique

**Figure 5:**
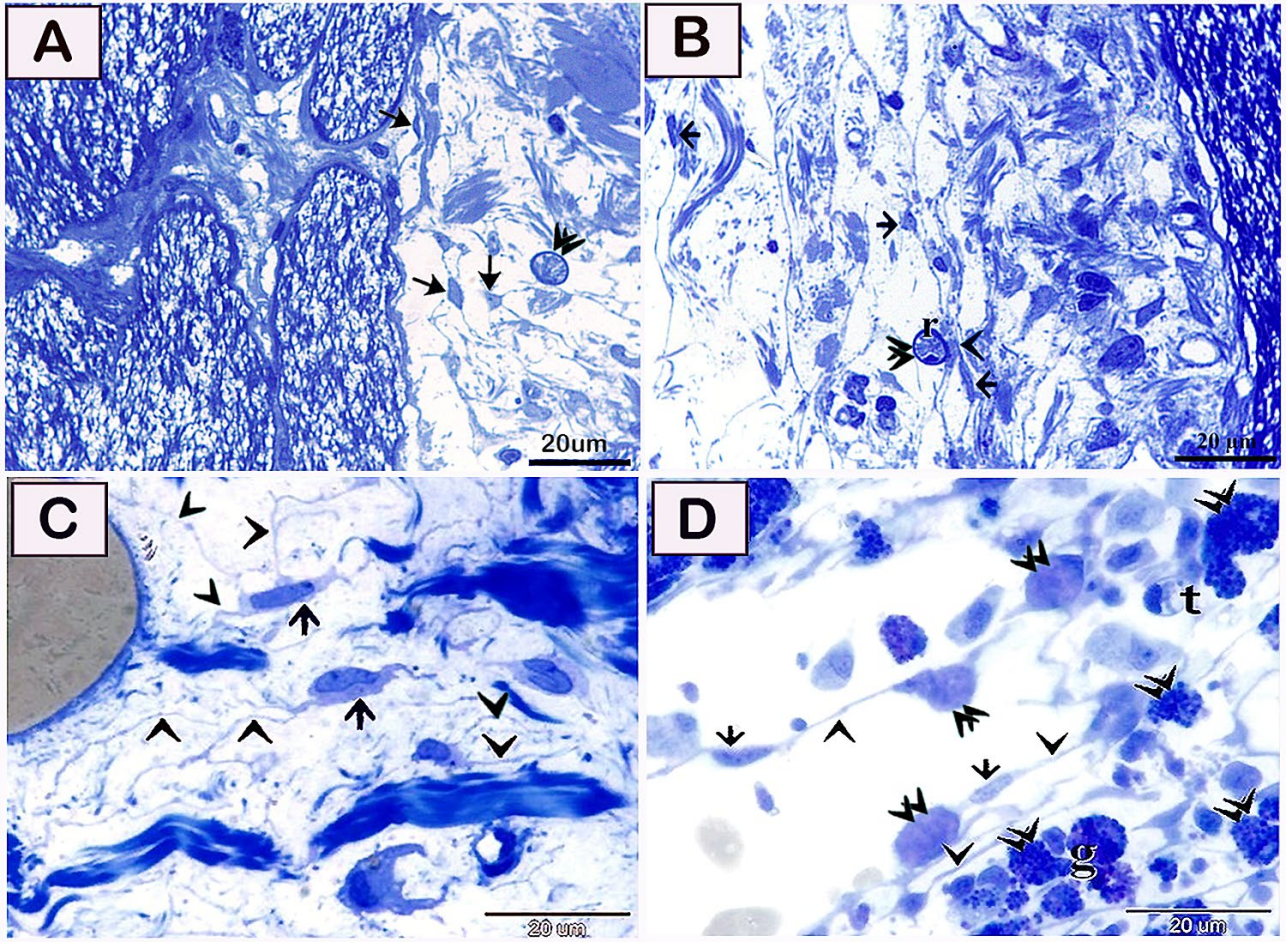
Telocytes in the olfactory organ and gills. (**A**,**B**) Semi thin sections in lamina propria of the olfactory organ stained by toluidine blue showing telocytes (arrows) in lamina propria and connected with transitional rodlet cells (r, double arrowheads). Note, telopodes (arrowheads). (**C**,**D**) Telocytes (arrows) and telopodes (arrowheads) formed a 3D network. Note rodlet cells (double arrowheads), granular rodlet cells (g), and transitional rodlet cells (t). Magnification: (**A**–**D**) ×1000.

Semi thin sections showed presence of telocytes in the olfactory organ (Fig. 5A,B), dermis of the skin (Fig. 5C), and gills (Fig. 5D). Lamina propria of the olfactory organ was rich in telocytes (Fig. 5A,B). Telocytes revealed the characteristic cell bodies and telopodes (Fig. 5C,D). They formed a network that encloses various stages of differential rodlet cells including granular rodlet cells and the transitional rodlet cells (Fig. 5A,B).

#### Immunohistochemical examination and acridine orange staining (fluorescent stain)

**Figure 6:**
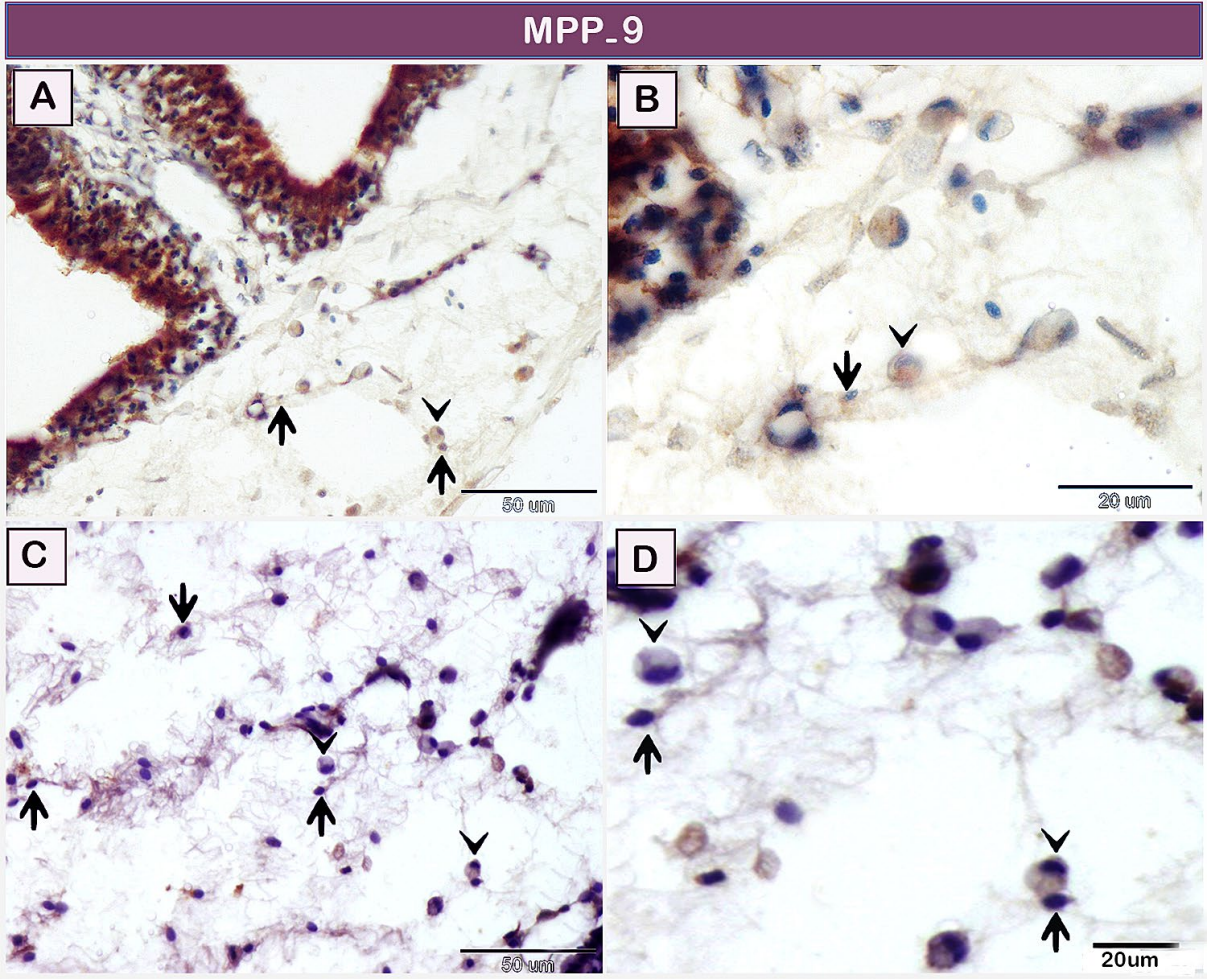
Immunohistochemical staining of the gill arch of the shark using MMP-9. Immunostained paraffin sections for MMP-9. Telocytes (arrows) express MMP-9 in the lamina propria of the olfactory organ (**A**,**B**) and the submucosa of the gill arch (**C**,**D**). Note rodlet cells (arrowheads). Magnification: (**A**,**C**) ×400; (**B**,**D**) ×1000.

**Figure 7:**
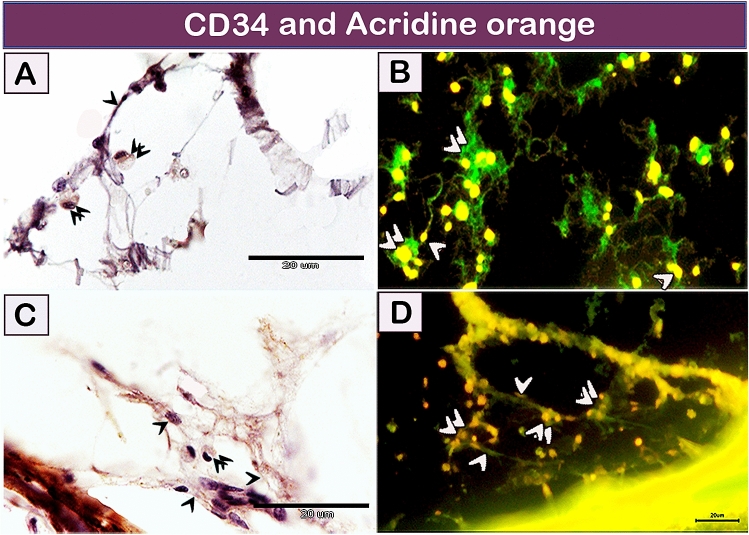
Recognition of TCs using CD34 and Acridine orange. (**A**,**C**) TCs (single arrowhead) were connected to rodlet cells (double arrowheads). (**B**,**D**) TCs formed a 3D network (single arrowhead) around rodlet cells (double arrowheads). Magnification: (**A**,**C**) ×1000; (**B**,**D**) ×400.

Telocytes within the lamina propria of the olfactory organ (Fig. 6A,B) and submucosa of the gill arch (Fig. 6C,D) exhibited positive immunoreactivity to MMP-9 metalloproteinases. The negative images for the MMP-9 metalloproteinases reactivity are presented in supplementary figure 1. Both telocytes (TCs) and rodlet cells were strong staining with antibodies against CD34 (Fig. 7A,C). The negative images for CD34 immunostaining are presented in supplementary figure 2. By using Acridine orange (AO) TCs formed a 3D network surrounding rodlet cells (Fig. 7B,D). Parallel figures for CD34 immunostaining and AO were used to demonstrate a similar view.

**Figure 8:**
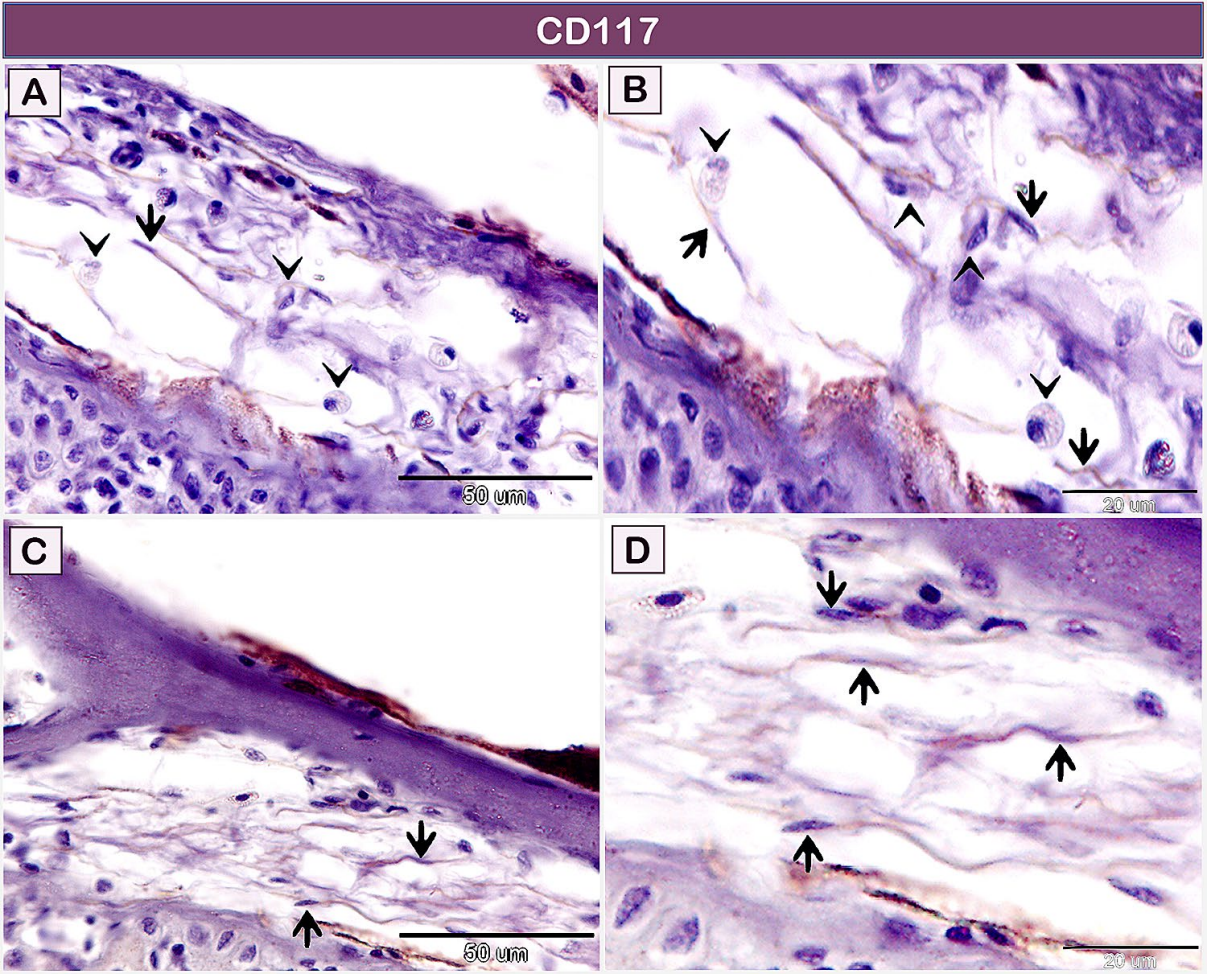
Immunohistochemical staining of the gill arch of the shark using CD117. Immunostained paraffin sections for CD117. Telocytes (arrows) in the submucosa of the gill arch express CD117. Note rodlet cells (arrowheads). Magnification: (**A**,**C**) ×400; (**B**,**D**) ×1000.

**Figure 9:**
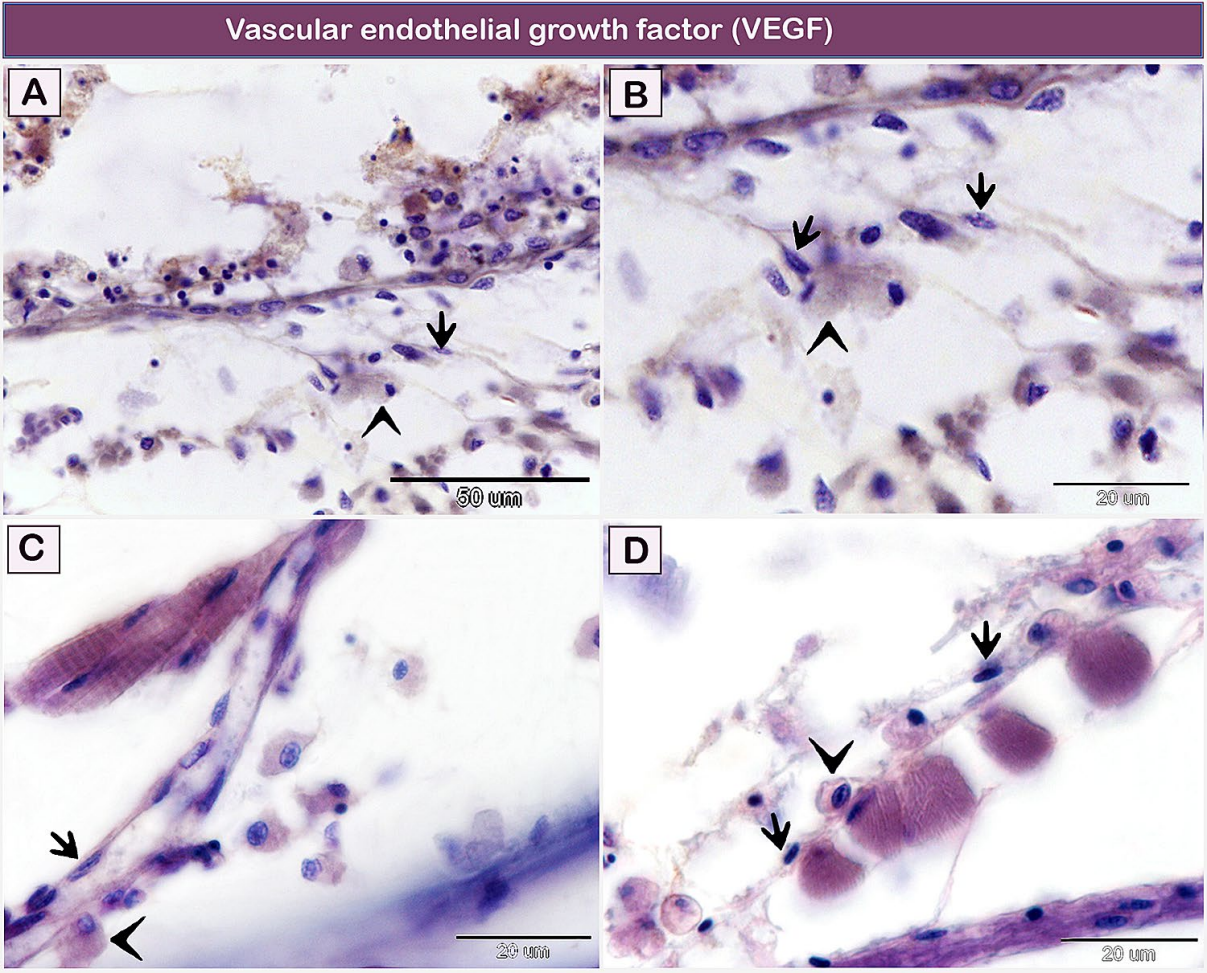
Immunohistochemical staining of the gill arch of the shark using VEGF. (**A**,**B**) Telocytes (arrows) express VEGF in the lamina propria. (**C**,**D**) Telocytes (arrows) express VEGF in the submucosa of the gill arch distributed between the skeletal muscles. Note rodlet cells (arrowheads). Magnification: (**A**) ×400; (**B**–**D**) ×1000.

CD117 immunostained TCs in the submucosa of the gill arch (Fig. 8A–D). The negative images for CD117 immunoreactivity are presented in supplementary figure 3. Telocytes in both the lamina propria (Fig. 9A,B), and the branchial arch muscles (Fig. 9C,D) revealed VEGF immunostaining. The negative images for VEGF are presented in supplementary figure 4. Data on the negative control of all markers are presented in supplementary figures 5 and 6.

### Electron microscopic investigation

#### Transmission electron microscopy examination

**Figure 10:**
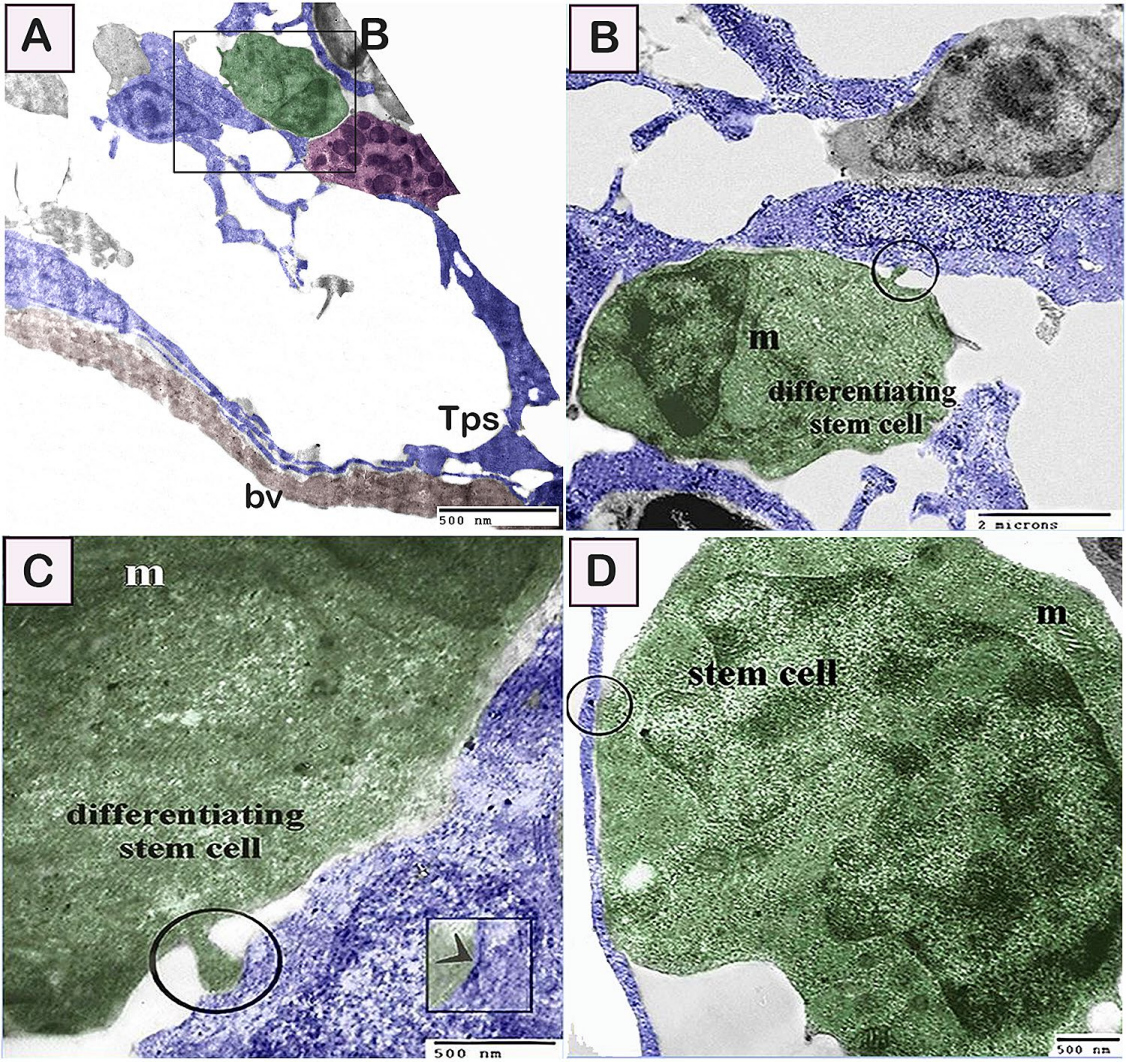
Relations between telocytes with stem cells. Colored ultra-thin sections in gill arches showing stem cells, various stages of rodlet differentiation. (**A**–**C**) Differentiating stem cells (green colored) acquired less nuclear/cytoplasmic ratio and elongated mitochondria (m). Note telocyte established contact with the finger-like process of stem cell. (circle). Note nanostructures (arrowhead in **C**). Note blood vessel (bv) and telopods (Tps). (**D**) A telopode (blue colored) established direct apposition contact (circle) with stem cell (green colored) which was identified by high nuclear/cytoplasmic ratio and had mitochondria (m). Magnification: (**A**) ×3600; (**B**) ×10,000 ; (**C**) ×29,000; (**D**) ×19,000.

**Figure 11:**
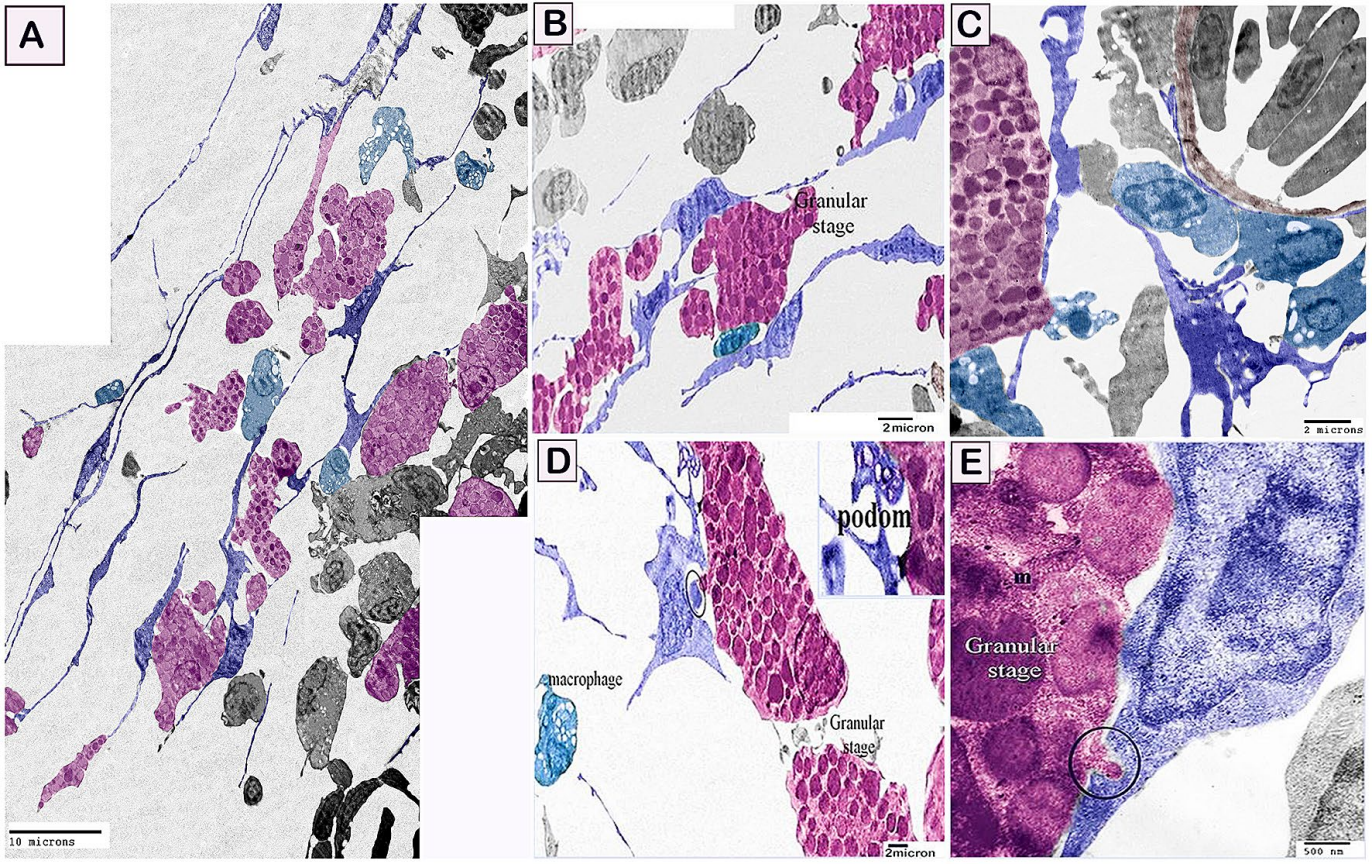
Relations between telocytes with granular stage of rodlet cells. (**A**–**D**) telocytes (blue color) forming a 3D interstitial network entrapped differentiating rodlet cells. Note granular rodlet cells (pink color). Podom established contact with granular rodlet cell. Telocytes establish direct contact (circle) with a cytoplasmic projection of the granular rodlet cell. Blood vessel (brown color). Monocytic cells (turquoise colored). (**E**) Finger-like cytoplasmic projection embedded in the telocyte cytoplasm (circle). Note mitochondria (m) and podom. Magnification: (**A**) ×1900; (**B**) ×3600; (**C**) ×4800; (**D**) X 4800; (**E**) ×19,000.

**Figure 12:**
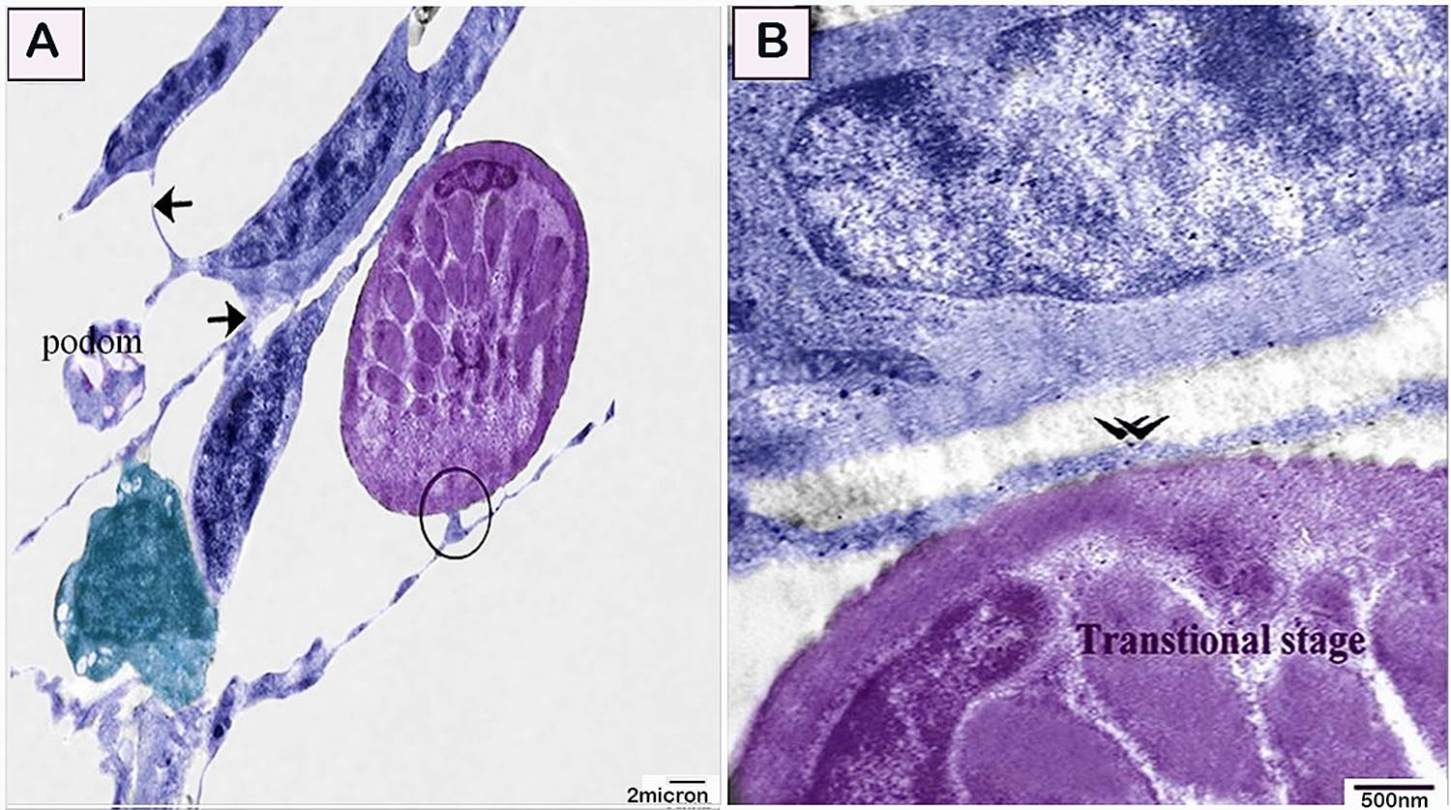
Relations between telocytes with the transitional stage of rodlet cell. (**A**,**B**) telocytes establish homocellular contact (arrows), and heterocellular contact (circle) with transitional rodlet cell (violet-colored, double arrowhead),) and macrophage progenitor (turquoise-colored). Note podom. Magnification: (**A**) ×4800; (**B**) ×19000.

By TEM, telocytes formed a 3D interstitial network entrapping different types of cells including the stem cells and differentiating cells (Figs. 10A, 11A–C). Stem cells are recognized to have a high nuclear/cytoplasmic ratio and presence of mitochondria, and telocytes establish direct contact with stem cells. Differentiating stem cells revealed less nuclear/cytoplasmic ratio and elongated mitochondria (Fig. 10B–D). In addition, differentiating stem cell and rodlet cells formed an interesting ultrastructure junctional modification to establish cell contact with telocytes. The established contact with the adjacent telocytes was formed through a finger-like process (Figs. 10A–C, 11E). Moreover, telocytes establish a direct contact with the cytoplasmic projection of the granular rodlet cell. Interestingly, podoms also established direct contact with granular rodlet cells (Fig. 11D,E). Telocytes established both homocellular contact (Fig. 12A), and heterocellular contact (Fig. 12B) with transitional rodlet cells and macrophage progenitor cells. Finger-like cytoplasmic projection embedded within the telocytes cytoplasm (Fig. 12A) was also noticed.

#### Scanning electron microscopy examination

**Figure 13:**
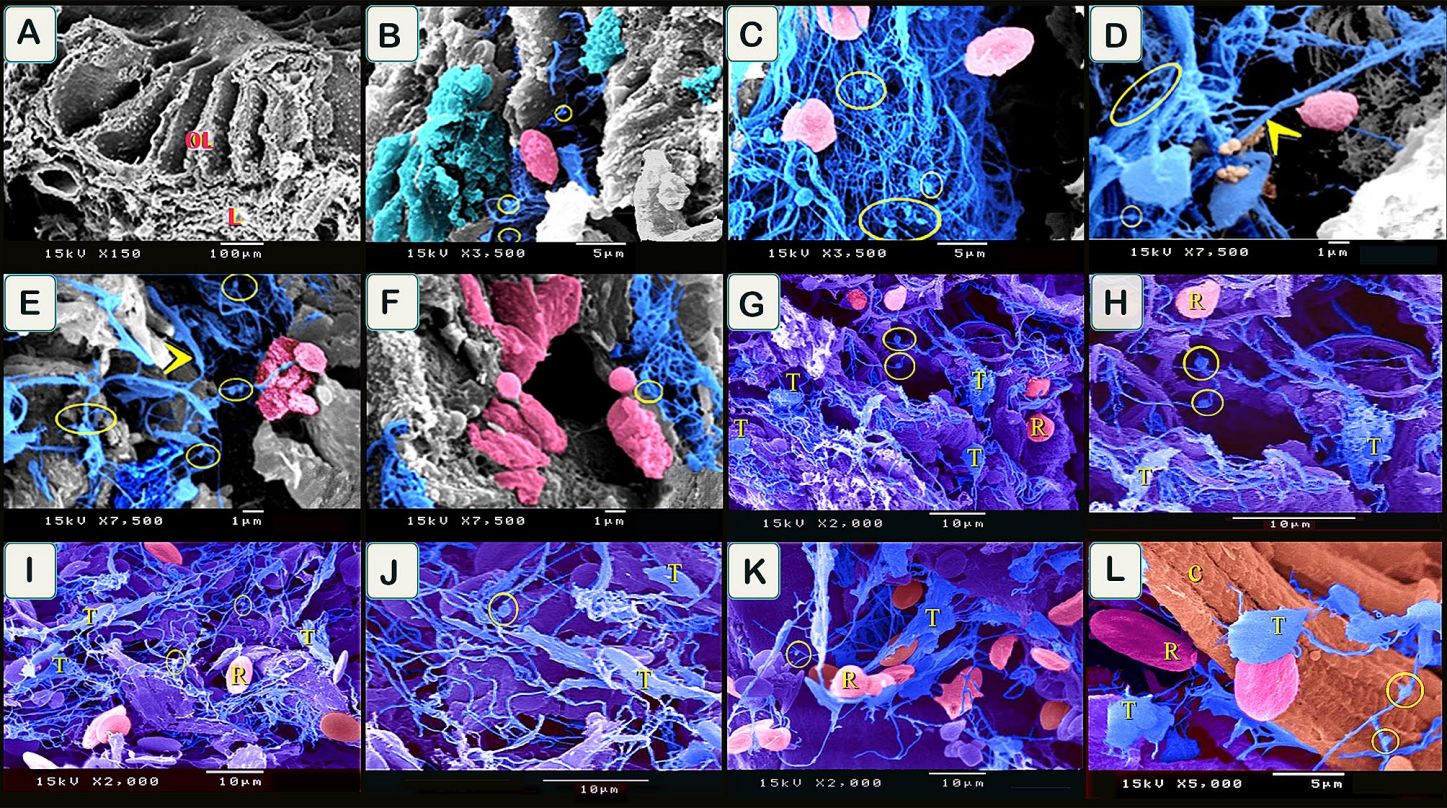
Scanned samples of olfactory organ. (**A**) general view of the olfactory organ. Olfactory lamellae (OL) and lamina propria (L). (**B**–**F**) telocytes (blue colored) established contact with granular rodlet cells (turquoise color), and transitional rodlet stage (pink colored). Note telopodes formed a 3D network around rodlet cells, yellow circle (podoms), podomers (arrowheads, telocytes secretion (brown color). (**G**–**J**) TCs (T) formed a 3D network in the lamina propria. Telopodes established contact with rodlet cells (R). Note podoms (yellow circle). (**K**,**L**) TCs (T) in the submucosa of the gill arch. They established contact with rodlet cells (R). Note podoms (yellow circle). Collagen fibers (C). Magnification on images.

**Figure 14:**
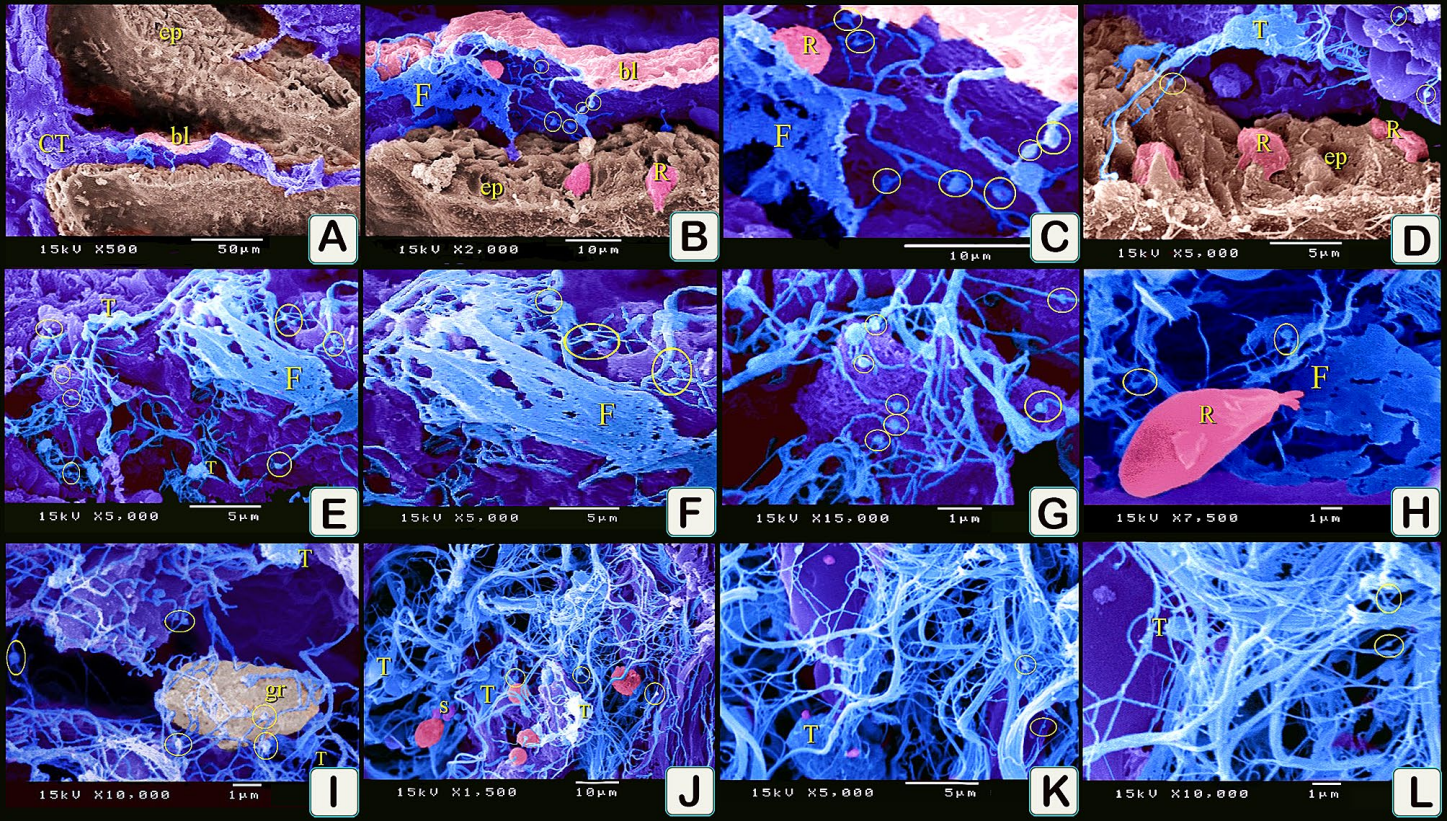
Scanned samples of olfactory organ. (**A**–**D**) olfactory organ lined by epithelium (ep), supported by connective tissue (CT). TCs (T) had an expanded telopodes to form fenestrated membrane (F). Note basal lamina (bl), podoms (yellow circle). Rodlet cells (R). (**E**–**L**) TCs (T) formed a 3D network in the lamina propria. Telopodes expanded to form fenestrated membrane (F). podoms (yellow circle), rodlet cells (R), granular rodlet cells (gr). Magnification on images.

Samples of the olfactory organ were examined using SEM (Fig. 13A). We identified typical telocytes that had podoms and podomers. Telocytes formed a 3D network around different stages of rodlet cells particularly at the granular and transitional rodlet stages (Fig. 13B–F). TCs formed a 3D network that established contact with rodlet cells in the lamina propria (Fig. 13G–J) and in the submucosa (Fig. 13K,L). TCs had an expanded telopodes to form fenestrated membrane in the olfactory organ, which made contact with rodlet cells (Fig. 14A–D). TCs formed a 3D network in the lamina propria where telopodes expanded to form the fenestrated membrane (Fig. 14E–L).

**Figure 15:**
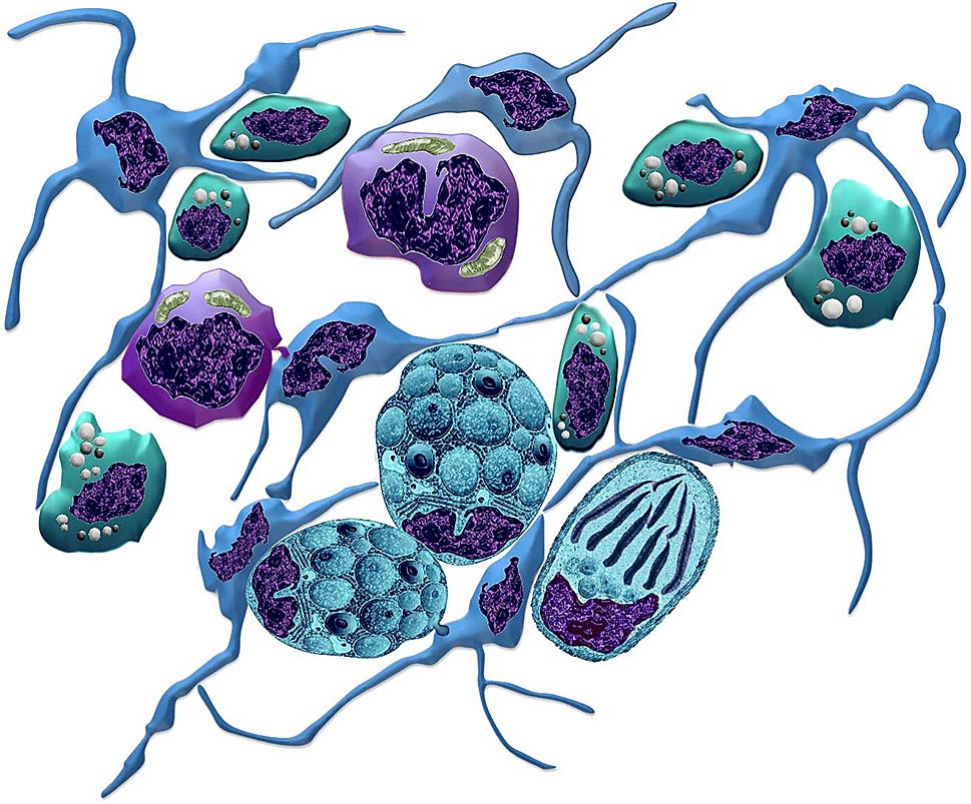
An illustration showing the relation of TCs and stem cells, stages of rodlet cells and macrophages. TCs (blue), stem cells (violet), rodlet cells (turquoise), and macrophage (green).

The relation of TCs with stem cells, rodlet cells, and macrophages was illustrated in Fig. 15.

## Discussion

The current investigation explored the correlation of telocytes with rodlet progenitor cells and other differential rodlet stages. We used histochemical staining, semi thin and ultra thin sections, and SEM to identify telocytes and rodlet cells.

Affinity for telocytes to different histochemical stains was studied using the Mallory triple trichrome, Crossomon’s trichrome, osmic acid, iron hematoxylin, Van Gieson staining, safranin O, silver, the Wiegert’s Van Gieson method , Sudan black, and AB/PAS. Telocytes exhibited a strong affinity for osmic acid and Sudan black B which may indicate staining of the plasma membrane phospholipids. Previous studies explored and discussed the telocyte affinity for Crossomon’s trichrome, AB/PAS, iron hematoxylin, Van Gieson, Safranin O, silver, Wiegert’s Van Gieson^31^. The authors have been documented the strong affinity of telocytes for PAS rather than Alcian blue^31^, in contrast to the observations of the current study. It seems that the nature of carbohydrate inclusions differs according to species or metabolic activity of the cells^73^.

The identification of TCs in the gill arch of the shark was confirmed by IHC. CD34 positive TCs were located in the lamina propria, the submucosa and between the branchial arch muscles. They formed a 3D network and established contact with rodlet cells. CD34 is a common marker for TCs in mammalian species^63,74–77^. Recently, CD34 positive TCs are identified in fish^78^. However, CD34 is a common transmembrane phosphoglycoprotein in hematopoietic stem cells. Moreover, it is detected in different types of progenitor cells including epithelial progenitors, muscle satellite cells, corneal keratocytes, interstitial cell progenitors, and vascular endothelial progenitors^79^.

Similarly, CD117 is used to identify TCs in mammals^31,79^. The CD117 protein is a transmembrane, tyrosine kinase growth factor receptor. The c-kit gene encodes the CD117 protein. Activation of CD117 regulates a wide variety of biological processes including apoptosis, cell differentiation, proliferation, chemotaxis, and cell adhesion^80^.

TCs were mainly located around blood vessels and mostly expressed VEGF. Vascular endothelial growth factor (VEGF) is a member of the platelet-derived growth factor family. VEGF has a critical role in angiogenesis^81^, vascular integrity^82^ and vascular permeability^83^. VEGF regulates vascular endothelial cadherin (VE-cadherin) adhesion between the endothelial cells. VE-cadherin has an essential role in the regulation of vascular permeability and leukocyte trafficking^84,85^. Rodlet cells have a potential role in fish immune defense. These cells circulate in the blood to reach different organs^21^. Therefore, we suggested that VEGF might be regulated in the trans-endothelial migration of rodlet cells.

Fish telocytes were morphologically consistent with those of other mammals, avian species, and amphibians^31,86^. In the current study, telocytes had telopodes and podoms. Telopodes formed homocellular and heterocellular contacts. Heterocellular contact was found to be with stem cells, rodlet, and macrophage progenitor cells.

A different hypothesis has been proposed for telocytes. Expression of telocytes to stem cell-specific markers suggests that telocytes are as a subclass of the undifferentiated mesenchymal cells^86^. However, the specificity of gene expression patterns of telocytes has been estimated to compare with that of mesenchymal cells and fibroblasts^16^. It has also been hypothesized that an alternative function of telocytes is that they may play a major role in the differentiation of stem cells. This speculation is based on a closed association between telocytes and stem cells niches and progenitor cells in various organs including the heart^87^, lung^95^, skeletal muscles^88^, skin^86^, liver^89^, blood vessels^90^. meninges, and choroid plexus^91^. Different modes of cellular interaction between telocytes and stem cells have been described. Telocytes provide a signaling system to establish an adequate microenvironment^14^.

In the current investigation, telocytes comprise a three-dimensional interstitial network in the olfactory organ and gill arch and are connected with different cells, stem cells, rodlets, and macrophage. They exhibited ultrastructure junctional modifications to form communicating sites through the nanostructures***.***

Telocytes established different forms of cell contact during the differentiation of the stem, rodlet cells and macrophages. Stem cell are identified to have high nuclear-cytoplasmic ratio and existence of mitochondria. During the process of differentiation, the nuclear-cytoplasmic ratio is reduced and mitochondria elongate which enable more activities required for cellular differentiation^92,93^.

Communication of telocytes with undifferentiated and differentiated stem cells may considered as a piece of evidence for the role of telocytes in stem cell differentiation guidance. We suggested that telocytes might provide adequate conditions for sufficient cell differentiation. We also speculated the role of telocytes in the regulation of the fish immunity through rodlet cells. The evidence on telocytes and stem cell communication shows a promising potential in tissue regeneration. Telocytes in skeletal muscles have multiple roles during a repair. They express proliferative marker Ki67, pluripotency marker Oct4, and vascular proliferation marker VEGF. Thus, telocytes may enhance cell proliferation and angiogenesis^88^. Telocytes have been implicated in cell propagation and suppress apoptotic cell death and fibrous tissue formation^17^. Telocytes may participate in hepatic regeneration via interaction with hepatocytes and stem cells. They may stimulate hepatocytes generation and activate hepatic precursor stem cells after partial hepatectomy^89^*.* Moreover, telocytes have been implicated in tissue homeostasis based on functional impairment of telocytes in the lung, stomach, and heart of systemic sclerosis patients^94^. Pulmonary telocytes provide a special type of direct intercellular communication with the putative stem cells. Telopodes form bridging nanostructures connecting to stem cells^95^. Telocytes may generate the appropriate conditions for putative stem and progenitor cells for differentiation. They direct cardiomyocyte progenitors in epicardial stem cell niches for differentiation^96^.

Rodlet cells have an immunological role against pathogens^97^. They contribut in the cell-mediated nonspecific immune response through the holocrine mode of secretion for protection against pathogens^97^. TCs form direct contact with various types of immune cells including lymphocytes, plasma cells, eosinophils, basophils, macrophages, and mast cells^5,31^ in mammals; dendritic cells,; and lymphocytes in fish^78^. TCs-immune cells form juxtacrine cell-to-cell signaling sites or chemical synapses. They also regulate the immune response through paracrine signaling. Uterine TCs play an essential role in the stimulation of the peritoneal macrophages that become activated and gain numerous pseudopodia and cytoplasmic secretory granules after co-culturing with TCs and release higher levels of cytokines such as TNF-α, IL1-R1, and IL-10, but not TGF-β1, IL-1β, IL-23α, and IL-18. These data revealed the possible role of TCs in the immunoregulatory and immunosurveillance mechanism in tissue^80^. The study suggests TCs have a potential role in the regulation of rodlet cell function.

Telocytes exhibited metalloproteinase activity including MMP-9. Metalloproteinase is essential for matrix degradation. Degradation of ECM is required for cell migration such that the ECM components act as physical barriers to hinder cell movement and invasion. Rodlet cells and macrophages are considered wondering cells. It seems thattelocytes express MMP-9 to facilitate immune cell movement and migration. Moreover, MMPP-2 and MMP-9 metalloproteinases are detected in telocytes in the gonads of Diplectrum formosum and Synbranchus marmoratus^97^. MMP-2 and MMP-9 are implicated in tissue remodeling of fish^98^.

In conclusion, telocytes have a potential role in regeneration that influences the stem/progenitor cells and the regulation of rodlet cell activities.

## Supplementary information

Supplementary Information.

## Data Availability

All data generated or analyzed during this study are included in this published article and its Supplementary Information files.
