# Publisher Correction: Fish telocytes and their relation to rodlet cells in ruby-red-fin shark (rainbow shark) *Epalzeorhynchos frenatum* (Teleostei: Cyprinidae)

**DOI:** 10.1038/s41598-021-89891-0

**Published:** 2021-05-31

**Authors:** Hanan H. Abd-Elhafeez, Walied Abdo, Basma Mohamed Kamal, Soha A. Soliman

**Affiliations:** 1 Department of Anatomy, Embryology and Histology, Faculty of Veterinary Medicine, Assiut University, Assiut, 71526 Egypt; 2 Department of Pathology, Faculty of Veterinary Medicine, Kafrelsheikh University, Kafr el‑Sheikh, 33516 Egypt; 3 Anatomy and Embryology Department, Faculty of Veterinary Medicine, University of Sadat City, Sadat City, 32897 Egypt; 4 Department of Histology, Faculty of Veterinary Medicine, South Valley University, Qena, 83523 Egypt

Correction to: *Scientific Reports* 10.1038/s41598-020-75677-3, published online 03 November 2020

This Article contained errors.

The version of this Article previously published quoted an incorrect email address for Hanan H. Abd-Elhafeez. Correspondence and requests for materials should also be addressed to hhnnzz91@aun.edu.eg

In addition, Affiliation 1 was incorrectly given as “Department of Anatomy, Embryology and Histology, Faculty of Veterinary Medicine, Assuit University, Assuit 71526, Egypt”.

The correct affiliation is listed below:

Department of Anatomy, Embryology and Histology, Faculty of Veterinary Medicine, Assiut University, Assiut 71526, Egypt.

Furthermore, the legend of Figure 1,

“Visualization of telocytes in the skin, intestine, head, and gill arch of the red-tailed shark using H&E.”

now reads:

“Visualization of telocytes in the skin, intestine, head, and gill arch of the red-fin shark using H&E.”

Lastly, in the Results section,

“The current study investigated the localization of telocytes in the red-tailed shark and their relation to stem cells and rodlet cells at different stages of development.”

now reads:

“The current study investigated the localization of telocytes in the red-fin shark and their relation to stem cells and rodlet cells at different stages of development.”

These errors have now been corrected in the PDF and HTML versions of the Article.

