# Supplementary material for: Fish telocytes and their relation to rodlet cells in ruby-red-fin shark (rainbow shark) Epalzeorhynchos frenatum (Teleostei: Cyprinidae)
Source: Sci Rep. 2020 Nov 3;10:18907. doi: 10.1038/s41598-020-75677-3 (PMC7641163; doi:10.1038/s41598-020-75677-3)
Supplement: Supplementary file 1 — Supplementary Information. [file 41598_2020_75677_MOESM1_ESM.pdf]

Supplementary file

Fish Telocytes and their relation to Rodlet Cells in Ruby-Red Fin Shark (Rainbow Shark) Epalzeorhynchos frenatum (Teleostei: Cyprinidae)

Hanan H. Abd-Elhafeez1 *, Walied Abdo2, Basma Mohamed Kamal3, Soha A. Soliman4

1 Associate professor of histology, Department of Anatomy, Embryology and Histology, Faculty of Veterinary Medicine, Assiut University, Assiut (71516), Egypt .Orcid.org/0000-0002-2547-0709

2 Associate professor, Department of Pathology, Faculty of veterinary medicine, Kafr el Sheikh University, Kafr El Sheikh.,33516, Egypt. Orcid.org/0000-0003-4635-8342

3Lecturer of anatomy, Anatomy and Embryology Department, Faculty of Veterinary Medicine, University of Sadat City, Sadat City, Egypt

4 Associate professor of histology, Department of Histology, Faculty of Veterinary Medicine, South Valley University, Qena 83523, Egypt. Orcid.org/0000-0001-6507-8405

CMEIAS color segmentation: (for the all supplementary images)

Negative images performed by using CMEIAS Color Segmentation(CMEIAS Color Segmentation is a free, improved computing technology), https://www.mybiosoftware.com/cmeias-color-segmentation-1-0-segment-analyze-foreground-objects-complex-images.html

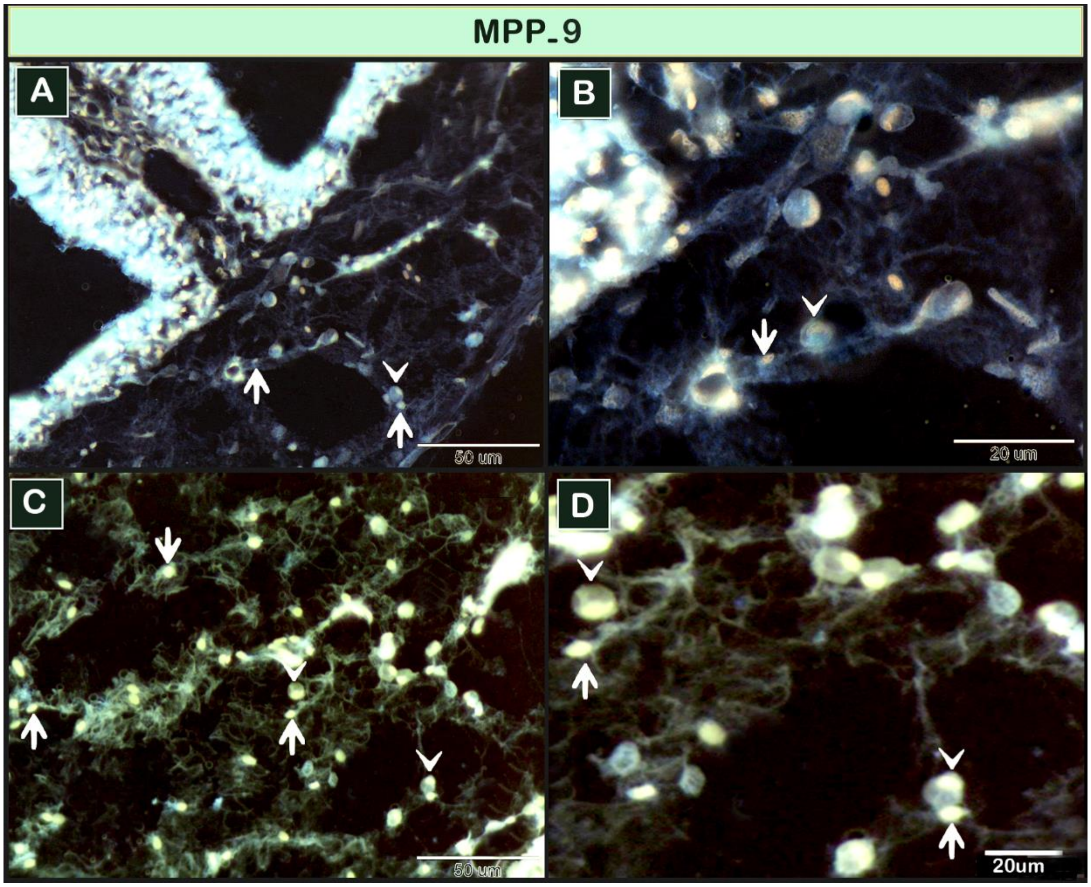

Fig 1: Negative image of immunohistochemical staining of the gill arch of the shark using MMP-9.

Immunostained paraffin sections for MMP-9. Telocytes (arrows) express MMP-9 in the submucosa of the gill arch. Note telopodes (arrowheads) formed 3D network.

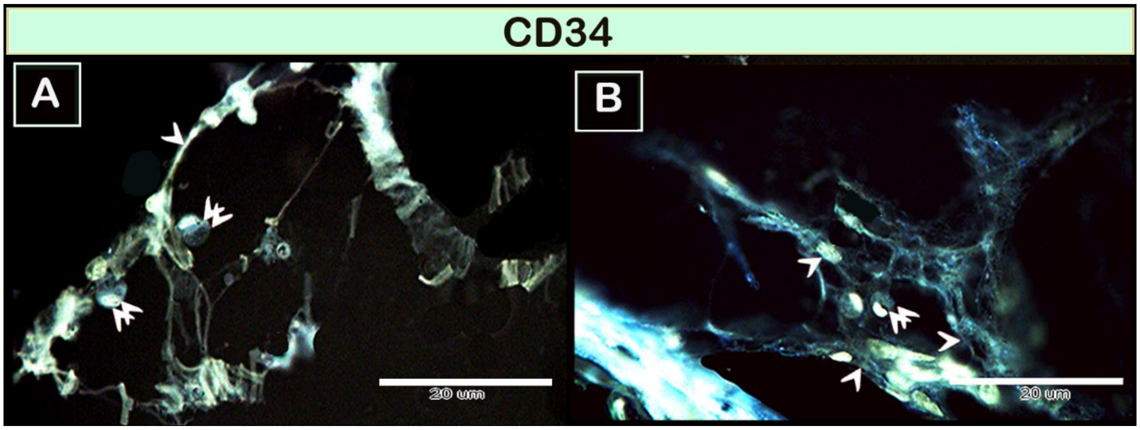

Fig 2: Negative image of immunohistochemical staining of the gill arch of the shark using CD-34. A, B: TCs (arrowheads) were connected to rodlet cells (double arrowheads).

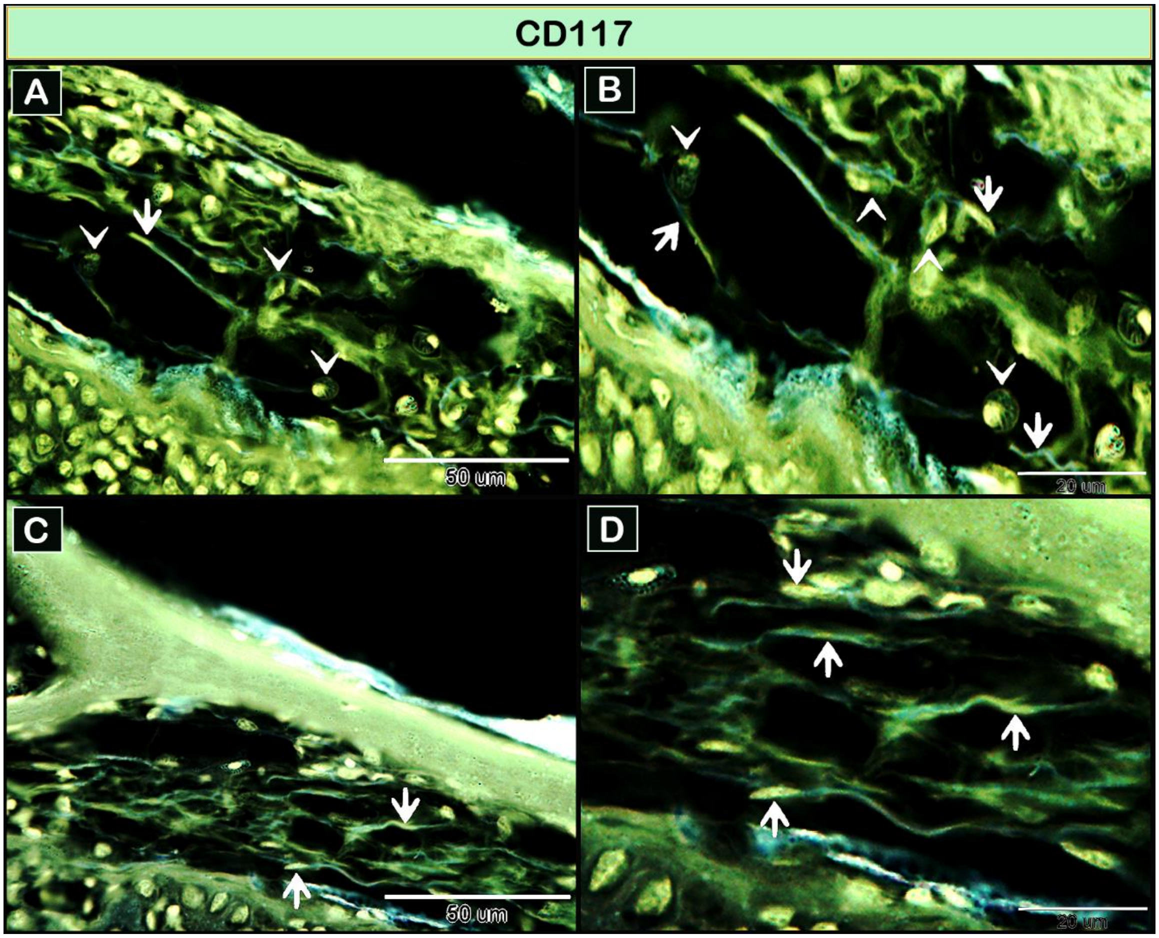

Fig.3: Negative image of Immunohistochemical staining of the gill arch of the shark Using CD-117

Immunostained paraffin sections for CD-117. Telocytes (arrows) in the submucosa of the gill arch express CD-117. Note rodlet cells (arrowheads)

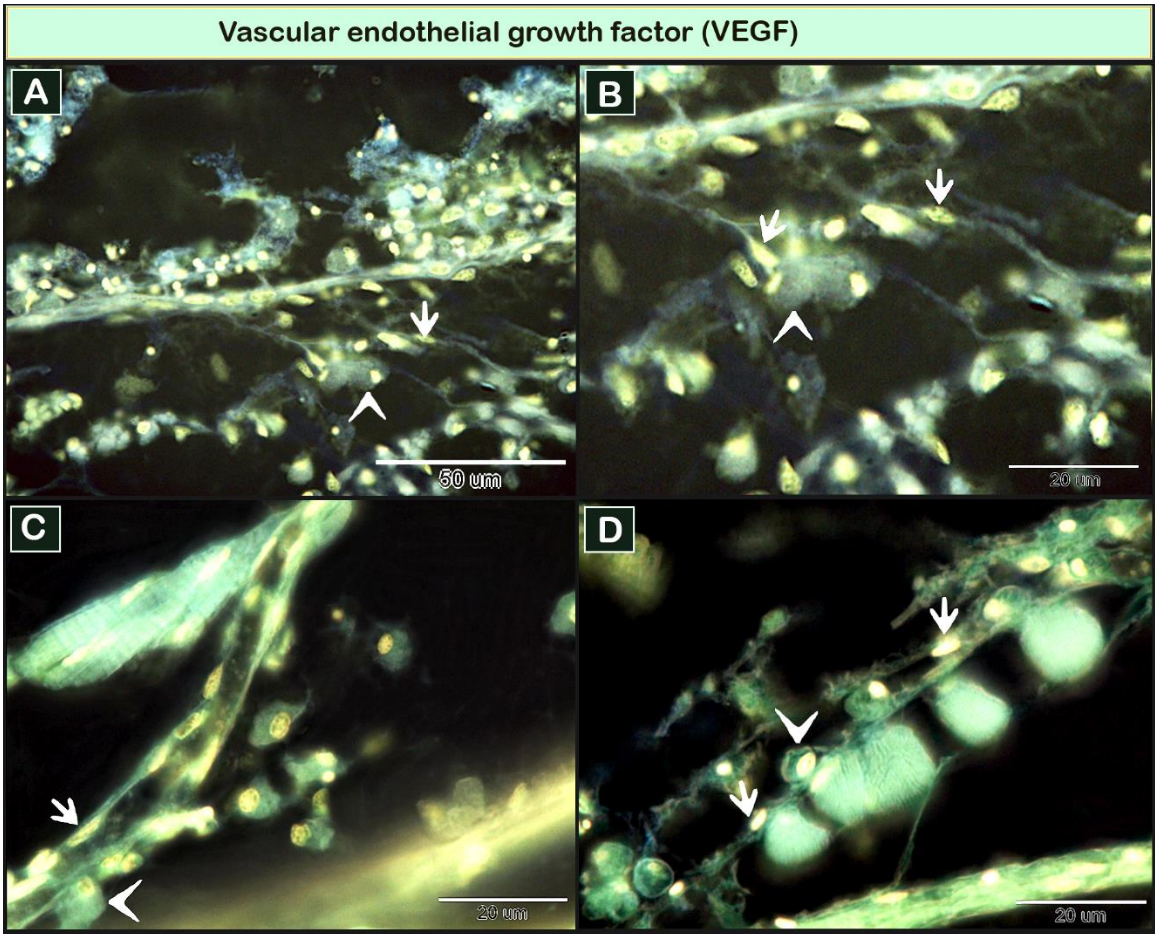

] Fig. 4: Negative image of immunohistochemical staining of the gill arch of the shark using VEGF.

A and B: Telocytes (arrows) express VEGF in the in the in the lamina propria. C and D: Telocytes (arrows) express VEGF in the submucosa of the gill arch distributing between the skeletal muscles. Note rodlet cells (arrowheads).

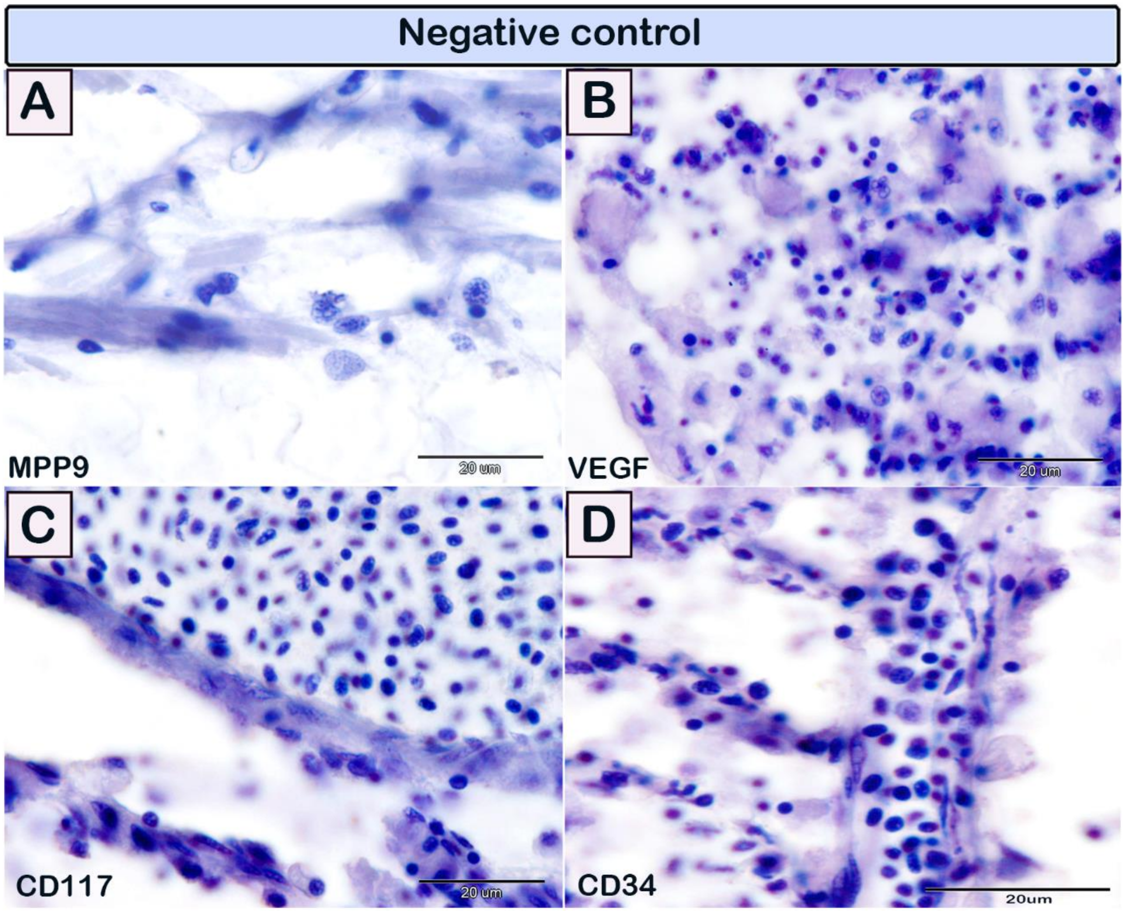

Fig. 5: Negative image of immunohistochemical staining of the gill of the shark Using A: MPP9, B: VEGF, C:, CD34, D: CD117.

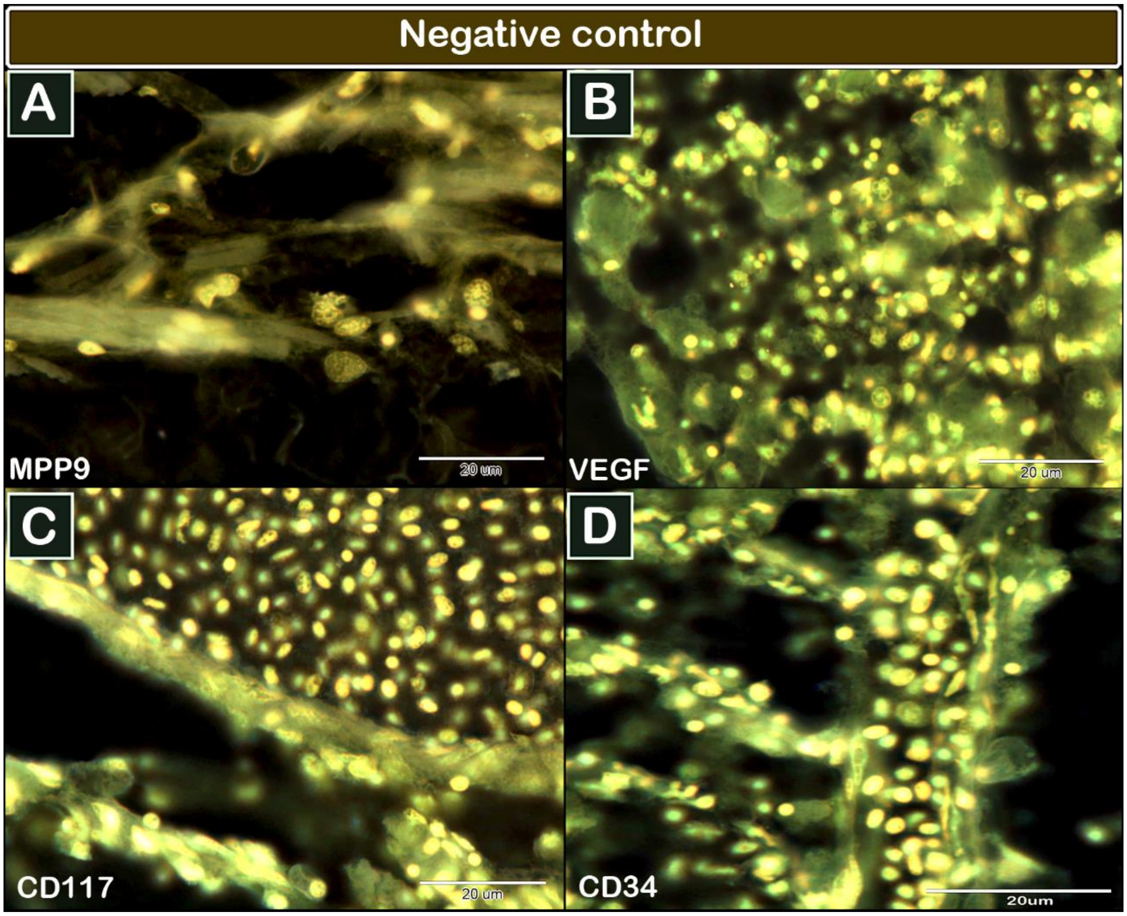

Fig.6: Negative image of negative control immunohistochemical staining of the gill of the shark Using A: MPP9, B: VEGF, C: CD34, D: CD117.

Note:

CD34, CD117, Mpp9, VEGF that showed reactivity in fish species according to the data sheet manufacture and publication by Abd-Elhafeez, H. H., et al. (1)

1-Abd-Elhafeez, H. H., et al. (2020) *Migratory activities and stemness properties of rodlet cells.* Microsc. Microanal. **21**: p. 1-18. .

2-
